# Phylogenetic Analysis of *NEAT1* and *MALAT1* Long Non-Coding RNAs Highlights Structure–Function Relationships in Paraspeckle Biology

**DOI:** 10.1093/molbev/msaf265

**Published:** 2025-12-09

**Authors:** Ksenia Arkhipova, Micha Drukker

**Affiliations:** Division of Drug Discovery and Safety, Leiden Academic Centre for Drug Research (LACDR), Leiden University, Einsteinweg 55, Leiden 2333 CC, The Netherlands; Division of Drug Discovery and Safety, Leiden Academic Centre for Drug Research (LACDR), Leiden University, Einsteinweg 55, Leiden 2333 CC, The Netherlands; Department of Internal Medicine, Leiden University Medical Center, Albinusdreef 2, Leiden 2333 ZA, The Netherlands

**Keywords:** phylogenetic, non-coding RNA, NEAT1, MALAT1, paraspeckle

## Abstract

Paraspeckles are nuclear bodies essential for gene regulation and stress response, and they are built upon the long non-coding RNA NEAT1. Together with the syntenic MALAT1, these are the only lncRNAs that use the tRNA-processing machinery for maturation, yet they differ in function and evolutionary conservation. To investigate these differences, we identified NEAT1 and MALAT1 orthologs across 545 mammals. For NEAT1, we found that G-quadruplexes, short motifs interacting with DBHS proteins and TDP-43, long gene length, and self-complementary regions are highly conserved features that likely stabilize paraspeckle integrity. Transposable elements also contributed structural modules potentially recognized by DBHS proteins, underscoring their role in NEAT1 evolution. The NEAT1Short isoform was present in all orthologs, and the TDP-43-mediated isoform switch appears to be conserved. In contrast, MALAT1 function likely relies on its conserved primary sequence and regions under purifying selection. This is the first large-scale phylogenetic study of *NEAT1*—a lncRNA that lacks sequence similarity between orthologs while maintaining functional and syntenic conservation.

## Introduction

Retrieving functionally important regions from an analysis of conservation patterns of primary and secondary structures of proteins and non-coding RNAs is a common approach. The method is based on the identification of conserved regions between orthologs, highlighting the pressure of purifying selection, which ensures that deleterious mutations are not established in the population, thereby maintaining only functionally essential structures (Charlesworth et al. 1993). Detailed mechanisms of function for the two long non-coding RNAs, *NEAT1* and *MALAT1*, connected by the uniqueness of their maturation processes, are not yet clear. However, the association of these genes with neurodegenerative diseases and cancer highlights the urgent need to identify regions and properties crucial for their function.

**Fig. 1.**
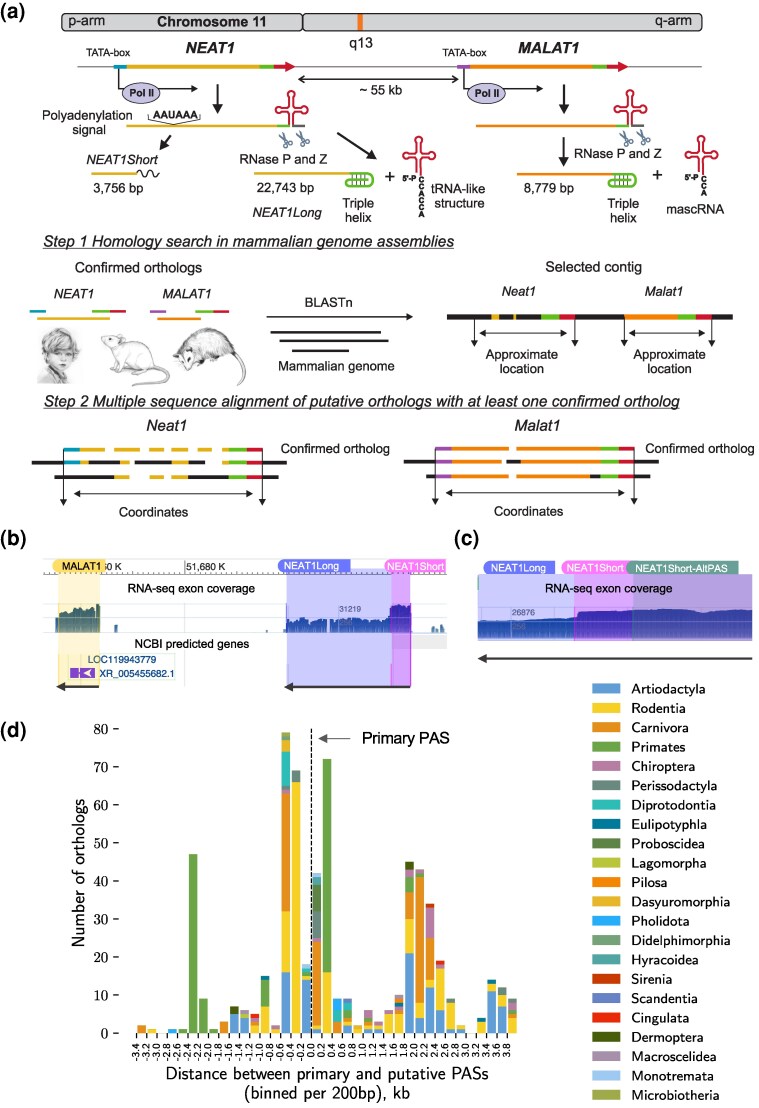
**Identification of *NEAT1* and *MALAT1* orthologs**. (a) The organization of *NEAT1* and *MALAT1* genes and the logic of orthologs' coordinates identification. Promoter areas, TATA-boxes, tRNA-like structures, and triple helices are highlighted, with colors used uniformly throughout the scheme. Genomic regions lacking sequence similarity to confirmed orthologs are depicted in black. (b) Confirmation of *NEAT1* and *MALAT1* ortholog predictions in *Tachyglossus aculeatus* (Monotremata order, short-beaked echidna)—the phylogenetically oldest species in our collection. Predicted coordinates were overlaid (shaded areas) on mapped transcriptomic read profiles in the Genome Browser (http://genome.ucsc.edu, Raney et al. 2024). In the “Genes” section of the Genome Browser, the automatically predicted genes identified in the region are shown. *Neat1* and *Malat1* are coded on the minus strand, with transcription direction indicated by arrows. (c) Two predicted PASs in *Tachyglossus aculeatus*. Zoom-in view on the transcription profiles of *Neat1* in *Tachyglossus aculeatus* near the 3′-end of the *Neat1*Short isoform. The coordinates of the main and alternative PASs are overlaid. The primary PAS corresponds more closely to the drop in transcriptomic reads. (d) Location and taxonomic distribution of alternative PASs in mammals. Most species possess an alternative PAS within 600 bp up- or downstream of the main PAS.

*NEAT1* and *MALAT1* share unique structural elements at their 3′-ends and the maturation processing machinery. Both genes are located on chromosome 11 in the human genome, positioned in close proximity to each other and coded on the same strand (Fig. 1a), with SCYL1 adjacent to *MALAT1* and FRMD8 bordering *NEAT1*. Similar localization of the genes in the mouse genome suggests possible synteny in other mammalian genomes as well (Stadler 2010). *NEAT1* is notably longer than *MALAT1*, spanning around 23 kilobases compared to *MALAT1*'s 8 kilobases. Similar to other lncRNAs, *NEAT1* and *MALAT1* are transcribed by polymerase II, but unlike any other known lncRNAs, tRNA-processing machinery is involved in their maturation. Specifically, the 3′-end of the genes forms a tRNA-like structure, which is recognized by RNase P and RNase Z, introducing two cuts before and after this structure, respectively (Fig. 1a) (Wilusz et al. 2008; Sunwoo et al. 2009). After the tRNA-like structure is cut out, the newly formed 3′-end folds into a triple helix, which stabilises the transcript (Brown et al. 2012). The tRNA-like structure (called mascRNA in the *MALAT1* gene) is further processed by another enzyme of tRNA maturation machinery—the CCA-adding enzyme, which can add two CCAs to the 3′-end of the structure instead of a single CCA, triggering its degradation. In five tested human cell lines it was demonstrated that *NEAT1*'s tRNA-like structures degrade in the cytoplasm, while mascRNA remains stable (Wilusz et al. 2008, 2011). The triple helix and tRNA-like structures of *MALAT1* are exceptionally conserved and have been detected across a wide range of vertebrates, including zebrafish, lizards, and reptiles (Stadler 2010; Zhang et al. 2017; Monroy-Eklund et al. 2023). Conservation of *NEAT1* tRNA-like structure has also been demonstrated among several mammals (Marz et al. 2014).

*NEAT1* encodes two isoforms, the long and the short (Fig. 1a), which we refer to as *NEAT1Long* and *NEAT1Short*, also known as *NEAT1*_1 and *NEAT1*_2, respectively (Naganuma et al. 2012). These isoforms share the 5′-end of the *NEAT1* gene, with the *NEAT1Short* undergoing polyadenylation at approximately 3.7 kb of the gene. To date, it remains an open question whether *NEAT1Short* exists in all mammalian species encoding *NEAT1*.

*NEAT1Long* is an architectural nuclear-retained RNA, which is an essential component of paraspeckles (Sasaki et al. 2009). These nuclear bodies are built around *NEAT1* and stabilized by proteins of two main classes. Members of the Drosophila behaviour/human splicing (DBHS) family (NONO, SFPQ, PSPC1) are multidomain oligomerizing proteins capable of binding nucleic acids (reviewed in Knott et al. 2016). NONO and SFPQ can also recognise secondary structures like stem loops, which can be formed from splice sites or inverted repeats of transposable Alu elements (IRAlu) or G-quadruplexes—guanine tracks separated by loops organized in layers by Hoogsteen hydrogen bonds (Knott et al. 2016; Simko et al. 2020; Mou et al. 2022). These proteins can form dimers with each other, enriching the diversity of interactions within paraspeckles. Another group of proteins that stabilize paraspeckles contain prion-like domains (Hennig et al. 2015). FUS and RBM14 are examples of essential paraspeckle proteins of this type (Hennig et al. 2015; Fox et al. 2018). The conservation and importance of the individual elements of *NEAT1* recognized by these proteins remain unclear. It is also uncertain how interchangeable these proteins are, as not all proteins in these families have been identified as essential in specific types of cells, with many considered merely important (Fox et al. 2018).

The formation of paraspeckles is linked to the transcription of *NEAT1* molecules. Initially, these molecules coalesce and then recruit multidomain proteins and other paraspeckle components (Mao et al. 2011). The paraspeckle structure consists of two main parts: the inner “core” and the outer “shell” (Hirose et al. 2019). These are distinguished by the folding of *NEAT1*, where the 3′ and 5′ ends are located in the “shell”, while the middle part of the gene forms the “core”, and by the predominant localization of resident proteins (Hirose et al. 2019). While the paraspeckle structure has been established in multiple cell types, the individual elements responsible for securing the distribution of resident proteins are less understood.

Current data about the conservation of *NEAT1* is inconsistent. In the early phylogenetic study on a diverse but limited set of around eight mammalian genomes, it was demonstrated that *NEAT1* orthologs are identifiable in Eutherians while absent in marsupials, likely due to incomplete assemblies (Stadler 2010). However, between human and mouse *NEAT1* orthologs, there are only a few patches of similarity, although both form functional paraspeckles. Thus, *NEAT1* is an example of an lncRNA where a lack of sequence similarity does not imply a lack of function, like some other lncRNAs (Pang et al. 2006). This discrepancy—expecting that functional conservation necessarily implies primary sequence conservation—was confirmed by the identification and functional confirmation of *Neat1* in opossum cells (marsupials), where traces of sequence similarity could be found in only 6% of the gene's length (Cornelis et al. 2016). The fourth mammal in which the *Neat1* gene has been identified and paraspeckles are confirmed is the naked mole-rat (Yamada et al. 2022), although, sequence homology of this ortholog was not analysed in detail. During our research, *NEAT1* orthologs were identified in koala and platypus genomes, as well as in several non-mammalian vertebrates, using a computational approach (Weghorst et al. 2024). The conserved secondary structure could explain the ability of *NEAT1* to form paraspeckles. However, a comparison of the secondary structures of mouse and human short isoforms of *NEAT1*, which include only the first ∼4 kb, revealed predominantly different patterns, with only some regions of similarity (Lin et al. 2018). Therefore, the fundamental question of what elements are essential for *NEAT1* function remains open.

*MALAT1* is one of the most highly expressed genes in human cells. Like *NEAT1*, it is a nuclear-retained lncRNA. *MALAT1* is located in speckles—another type of nuclear body in close proximity to paraspeckles. Unlike *NEAT1*, *MALAT1* is a highly conserved lncRNA, with orthologs identified in zebrafish and other vertebrates (Stadler 2010; Weghorst et al. 2024). Moreover, the conservation of a large part of the *MALAT1* secondary structure was demonstrated in 51 mammals (McCown et al. 2019).

In this study, we aimed to investigate the structure–function axis of *NEAT1* and *MALAT1* by identifying conserved regions, sequence features, structures, and regulatory elements within a new large collection of orthologs from 545 diverse mammals. Our prediction of *NEAT1Short* isoforms and alternative polyadenylation signals (PAS) underscores the universal presence of the short isoform across all orthologs examined. We also analysed the conservation of transcriptional regulation, triple helix elements, and tRNA-like structures, further consolidating previously known findings. After analysing the overall diversity of *NEAT1* orthologs, we selected 16 the most dissimilar ones, which we called archetypes, for the identification of shared features. The primary sequence of the orthologs was scrutinized for nucleotide composition and the presence and enrichment of various repeats, like transposable elements (TEs) and short sequence motifs. Our analysis revealed the ubiquitous features likely most critical to paraspeckle function, including GU repeats, recognized by TDP-43, and G-quadruplexes. We identified specific patterns of TEs integration and their role in the evolutionary shaping of *NEAT1* and its function. Overall, our results suggest that certain domains, elements, structures, and RNA processing events in NEAT1 are universally crucial for the function of paraspeckles.

## Results

### Defining Genomic Coordinates for *NEAT1* and *MALAT1* Orthologs in 545 Mammals

Although *NEAT1* orthologs have been reported in a limited number of mammalian species and vertebrates (Stadler 2010; Cornelis et al. 2016; Weghorst et al. 2024), the nucleotide sequences available to us at the start of our study were for only three species: human, mouse, and opossum. With these three dissimilar sequences of the *NEAT1* gene available, we undertook the challenge of identifying *NEAT1* orthologs in mammalian genomic assemblies (Fig. 1a). The developed algorithm relied on synteny of *NEAT1* and *MALAT1*, as well as the high degree of conservation of *MALAT1.* We first searched for orthologs of *MALAT1*. Then, the homology patches of *MALAT1* served as anchoring points for genomic contig selection, and the surrounding regions were explored to locate *NEAT1*. Due to the considerable length of *NEAT1* gene, we separately searched for similarities to fragments containing the TATA-box of the promoter region and the triple helix followed by a tRNA-like structure. The outstanding high degree of conservation of these structures allowed us reliably identify 5′- and 3′- ends of *NEAT1* orthologs, despite the variability in the primary sequence of the gene. We reconstructed 506 *NEAT1* and 469 *MALAT1* gene orthologs (Figure S1a). In total, the identified *NEAT1* and *MALAT1* orthologs originate from 545 mammalian genomes (487 species, 122 families, 24 orders; Figure S1a), 17 of which belong to four orders of marsupials.

To substantiate the gene predictions, we inspected profiles of mapped transcriptomic reads using Genome Browser (Raney et al. 2024, Fig. 1b and c, Figure S1b). We input the established coordinates of both genes, including the short isoform(s), and compared our predictions with the results of transcriptome read mapping. We performed this verification for the most divergent *NEAT1* orthologs (archetypes), for which Genome Browser data were available (Figure S1b), and observed a very good agreement between our predictions and the expression profiles of both genes. Since the remaining orthologs exhibit clear sequence homology to at least one of the archetypes, we assumed that the transcriptomic read mapping pattern would be comparable. To further support our findings, we compared our results to *NEAT1* and *MALAT1* orthologs from the naked mole-rat (Yamada et al. 2022) and koala (Weghorst et al. 2024), with which there was very good agreement (see Methods).

One of the open questions about *NEAT1* is whether a short isoform is present and expressed in other mammals. We attempted to identify *NEAT1Short* isoforms in the orthologs by searching for the positions of the canonical polyadenylation signal (PAS), which comprises an “AATAAA” motif, and successfully identified a single PAS in all Eutherian. We noted that *NEAT1Short* forms a recognizable, twice-higher pattern in transcriptomic profiles (Fig. 1b and c), which we also observed in the phylogenetically oldest mammal in our collection, *Tachyglossus aculeatus* (order Monotremata, short-beaked echidna, Fig. 1c).

In the opossum (marsupial), Cornelis *et al*. found evidence for two active PASs approximately 500 bp apart (Cornelis et al. 2016), both of which we identified in all marsupials and Monotremata (Fig. 1c). Next, we asked whether an alternative PAS can be found in Eutherians as well. We showed that many orthologs (*n* = 275, 55%) have alternative PASs in close proximity, located on both sides of the “main” PAS (±600 bp, Fig. 1d) and the position of an alternative PAS is taxon-specific (Fig. 1d). Thus, *NEAT1Short* appears to be a ubiquitous mammalian isoform, with evidence for additional alternative isoforms in its vicinity.

Our search algorithm relied on the synteny between *NEAT1* and *MALAT1*. Indeed, 92% of the 428 mammalian genomes containing both *NEAT1* and *MALAT1* had the genes on the same contig. The genes were consistently encoded in close proximity, with the intergenic distance rarely exceeding 60 kb and averaging 36,755.3 ± 9,927.91 bp (Figure S1c). With the exception of two species (*Rousettus madagascariensis* and *Oryctolagus cuniculus*), both genes were encoded on the same strand of DNA. We suspect that the assembly quality may explain this observation, as neither of these assemblies belonged to the GenBank reference set. Overall, we successfully identified large set of *NEAT1* and *MALAT1* orthologs across mammalian taxa for phylogenetic analysis.

### Conservation of the Triple Helix and tRNA-like Structures of *NEAT1* and *MALAT1*

**Fig. 2.**
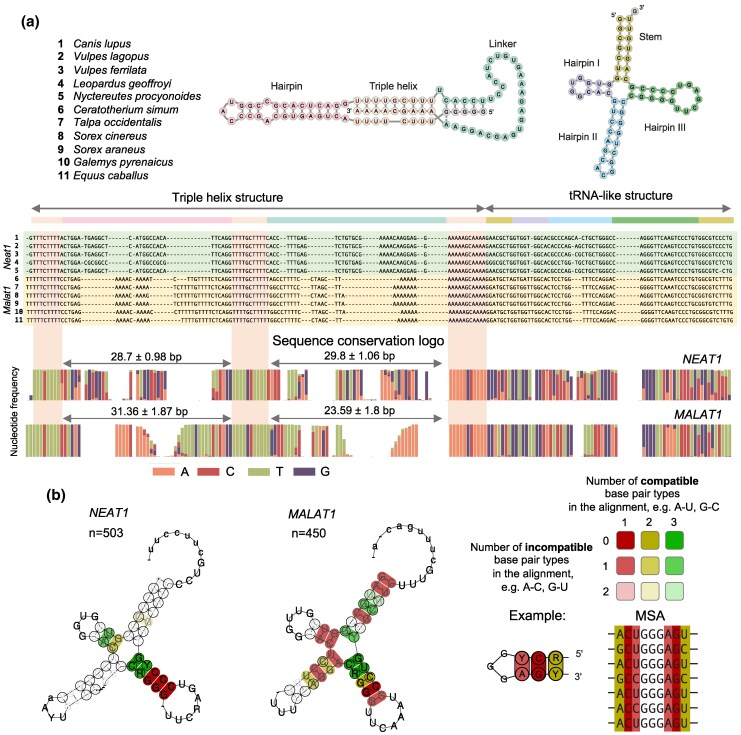
**Conservation of 3′-end motifs of *NEAT1* and *MALAT1* orthologs.** (a) Secondary structure and sequence diversity of triple helices and tRNA-like structures in *NEAT1* and *MALAT1* orthologs. Secondary structures of the human triple helix and tRNA-like structure are shown at the top of the figure, with individual structural elements highlighted. Colors are used consistently throughout the figure. An example of the multiple sequence alignment of 3′-end structures of both *NEAT1* and *MALAT1* orthologs from the listed randomly selected species is depicted. The summary of sequence diversity across all orthologs is presented as a colored sequence conservation logo. The variance in length (mean ± std) of hairpins I and II of triple helices in *MALAT1* and *NEAT1* orthologs is specified. Highly conserved triple helix-forming sequence regions are highlighted in both the alignment and logo figures. (b) Co-evolving patterns of tRNA-like structures across all *NEAT1* and *MALAT1* orthologs in Eutherians. The most conserved base pairs are shown in dark red. High-intensity yellow and green indicate perfectly matching alternative base pairs (co-evolving) in the MSA. The co-evolving patterns of the tRNA-like structure of *MALAT1* exhibit a much higher level of conservation in the whole secondary structure, while the tRNA-like structure of *NEAT1* mainly involves hairpin III with a highly variable hairpin II.

Next, we focused on the 3′-end elements—the triple helix and tRNA-like structures. While these structures of *MALAT1* are known to be highly conserved (Zhang et al. 2017), the situation for *NEAT1* was less clear. Overall, we found low divergence between the 3′-ends of both, *NEAT1* and *MALAT1,* orthologs across all mammals (Fig. 2a). The triple helix structure consists of three principal parts: the structure-forming motif itself, a hairpin loop and a linker (Fig. 2a). We found that the conservation of the structure-forming motif was exceptional, with no mismatches in any *NEAT1* or *MALAT1* ortholog (Fig. 2a). However, the sequences of the hairpin loop and the linker displayed clear specificity for *NEAT1* or *MALAT1* and had high sequence variability in *NEAT1*. In *NEAT1* orthologs, they were nearly equal in size (28.7 ± 0.98 bp and 29.8 ± 1.06 bp), whereas the linker of *MALAT1* was one-third shorter (31.36 ± 1.87 bp and 23.59 ± 1.8 bp, Fig. 2a). Therefore, our results suggest that, for *NEAT1* and *MALAT1* RNA stability, the sequence of the triple helix-forming motif is the most crucial element—possibly along with the length of the hairpin and the linker.

The conservation degree of the tRNA-like structures of *NEAT1* and *MALAT1* orthologs was high, although *NEAT1* orthologs exhibited slightly greater variation (Fig. 2a). We also analysed patterns of coordinated nucleotide changes in complementary pairs (co-evolving) to assess the pressure of purifying selection on the secondary structure of the tRNA-like elements. We found that the secondary structures of both genes are well conserved (Fig. 2b), and the sizes of individual elements, such as hairpin loops, did not vary drastically. Our analysis clearly highlighted the strongest purifying selection on the third hairpin loop of the tRNA-like structures in both genes, suggesting it has higher functional importance. Taken together, the high degree of sequence conservation of these structural elements highlights their critical role in processing and maturation of *NEAT1* and *MALAT1*.

### Analysis of Promoter and Transcriptional Control of *NEAT1* and *MALAT1*

**Fig. 3.**
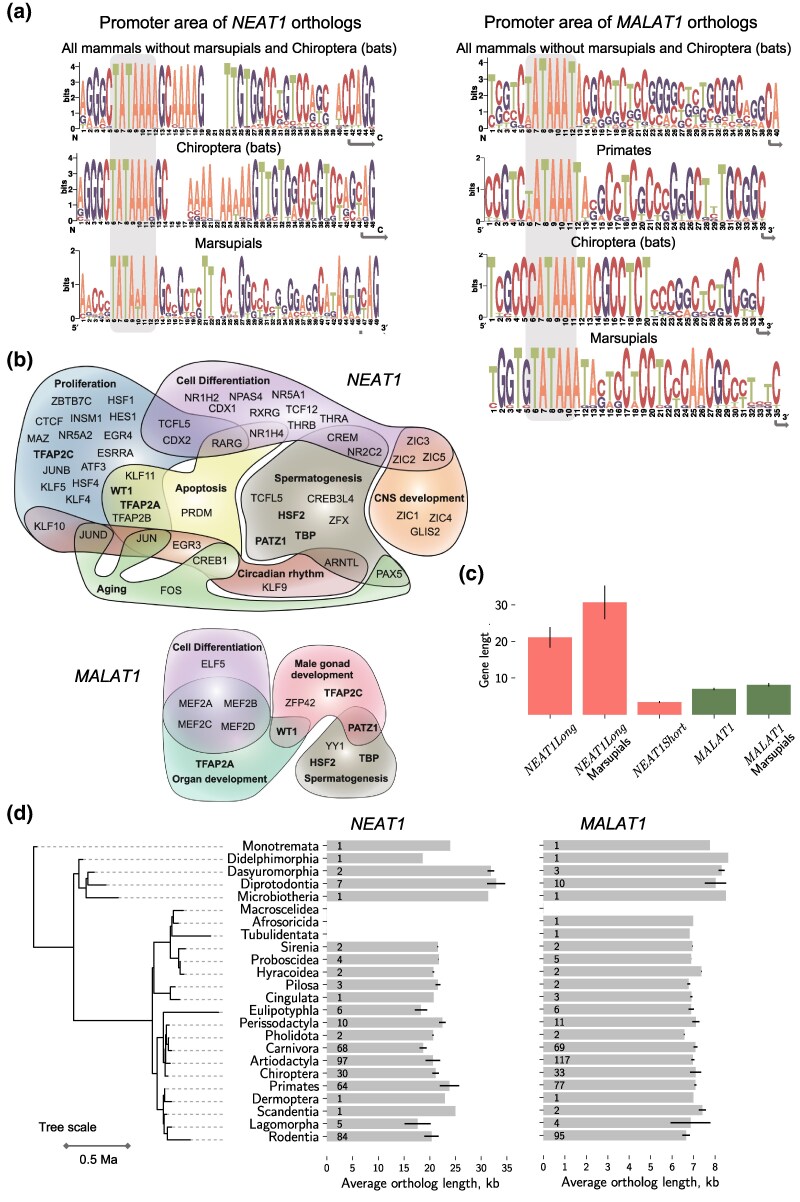
**Transcriptional regulation of *NEAT1* and *MALAT1* orthologs and their length distribution.** (a) Conservation of TATA-boxes and promoter areas in *NEAT1* and *MALAT1* orthologs (sequence logo). TATA-boxes are highlighted with grey boxes, and the transcription start site is marked by an arrow. (b) The most frequent GO terms associated with transcription factors, the binding sites of which were identified in at least 65% of orthologs of *NEAT1* and *MALAT1*. Shared biological processes associated with the same TF are depicted as overlaps. (c) Average ortholog length and its variation across mammals. Marsupials exhibit the longest average length for *Neat1* and *Malat1*, while the *NEAT1Short* isoform shows much smaller length variation compared to *NEAT1Long*. Only non-gapped ortholog assemblies are taken into account. (d) Length distribution of *NEAT1* and *MALAT1* orthologs in mammalian orders, arranged along a time-scaled phylogenetic tree. Only orthologs with non-gapped gene assemblies were used. The number of orthologs used for the assessment is indicated on the bars.

The conservation of promoter regions and transcription factors’ binding sites across species can highlight the importance of a gene within certain physiological processes. Conversely, variability in transcriptional regulation can suggest functional differences. We began with an analysis of the TATA-box and the downstream promoter area. Overall, this region was more conserved in *NEAT1* orthologs than in *MALAT1*, which is surprising given the opposite, greater primary sequence variability of *NEAT1* compared to *MALAT1* (Fig. 3a). We found that *NEAT1* orthologs in all Eutherians possessed the classical TATA-box sequence “TATAAA”, with greater promoter area diversity observed in marsupials. The variability of the transcription initiation site in *MALAT1* was significantly higher, and it was less variable only within individual mammalian taxa (e.g. Primates, Chiroptera in Fig. 3a). We also noted a higher diversity of TATA-box motifs in *MALAT1*, such as “CATAAA” in the Chiroptera order, and both “AATAAA” and the classical “TATAAA” in Primates.

As a next step, we predicted transcription factor (TF) binding sites within the 1 kb promoter area of *NEAT1* and *MALAT1* orthologs. An individual promoter of *NEAT1* and *MALAT1* orthologs had, on average, 216.4 ± 33 and 168.2 ± 29 TF binding sites, respectively. Although the average number of sites did not differ drastically, we investigated how many of these sites were identified between orthologs. Surprisingly, we observed that only a small number of TF binding sites were shared among the promoters of *MALAT1* orthologs. We applied a rather permissive threshold of 65% of orthologs per gene, resulting in 25 TFs for *MALAT1* and 123 TFs for *NEAT1* (Table S1). Among the predicted TF binding sites for *NEAT1* and *MALAT1* orthologs, we identified 15 that overlapped, including EGR1 and SP1, which have been experimentally validated (Li et al. 2015; Che et al. 2021; Kumar and Mishra 2022; Binder et al. 2023; Tian et al. 2023). Additionally, analysis of GO terms suggested regulation by transcription factors associated with the processes many of which have been experimentally validated for both genes (Fig. 3b) supporting the findings of this unique analysis. Overall, our results indicate a higher degree of conservation of the regulatory elements of *NEAT1* transcription compared to *MALAT1*.

### Gene Length Variation of *NEAT1* Orthologs

*NEAT1* is one of the longest known lncRNA in the human genome (Derrien et al. 2012), and its length may be a crucial parameter for its architectural function in facilitating phase separation and stabilizing paraspeckles. However, the length of two studied lncRNAs have not been a primary focus in previous studies. We analysed the distribution of lengths of *NEAT1* orthologs and found that the average length was 21,114.1 ± 2,811.3 bp (only assemblies without gaps were used). However, the difference between the longest and shortest variants was more substantial: 14,505 bp in *Ochotona curzoniae* (plateau pika, Lagomorpha) and 36,456 bp in *Gymnobelideus leadbeateri* (Leadbeater's possum, Diprotodontia). Notably, the lengths of *NEAT1Short* isoforms varied within a much narrower range, 3,415.18 ± 218.9 bp (Fig. 3c), which suggests potential functional importance.

We observed that the length of the *NEAT1Long* isoform and its variation exhibited some taxon-specific patterns (Fig. 3c and d). Marsupials from the Microbiotheria, Diprotodontia, and Dasyuromorphia orders had the longest *Neat1* genes of all mammals, averaging 30,659.9 ± 4,575.1 bp. However, we did not find evidence for a general evolutionary trend of *NEAT1* shortening as an association between gene length and the phylogenetic distance of a species from *Tachyglossus aculeatus*, Monotremata was not pronounced (Spearman's rho = −0.06, *P* = 0.18). Additionally, *NEAT1* length varied more within some orders, such as Primates and Artiodactyla, compared to Carnivora. The length of *MALAT1* orthologs varied within a narrower range than that of *NEAT1*, 6,986.8 ± 326.78 bp (Fig. 3d), with a taxon-specific pattern. Marsupials, like *NEAT1* orthologs, had the longest *Malat1* gene (8,124.25 ± 449.08 bp), while rodents exhibited the shortest *Malat1* gene (6,653 ± 176.3 bp). Our findings indicate that the exceptional length of *NEAT1* is conserved across mammals, implying a functional role in paraspeckle biology.

### *NEAT1* and *MALAT1* Orthologs Primary Sequence Diversity and *NEAT1* Archetypes

**Fig. 4.**
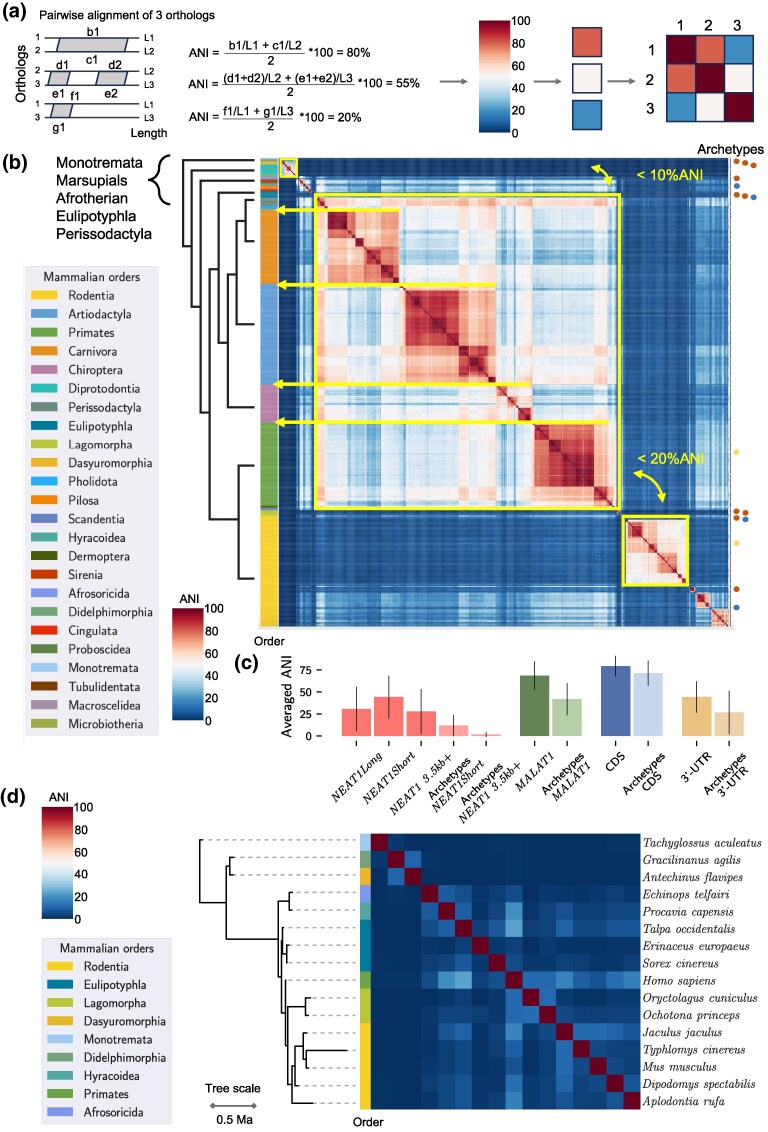
**Primary sequence diversity of *NEAT1* and *MALAT1* orthologs in mammals.** (a) Schematic representation of how average nucleotide identity (ANI) was calculated between pairs of genes and visualized as heatmaps. Patches of similarity from pairwise blastn alignments were normalized to the length of individual genes, averaged for both, and expressed as a percentage (see Methods). The obtained percentages were assigned corresponding colors and plotted in an all-to-all heatmap. (b) Heatmap of ortholog similarity in pairwise comparisons (all-to-all). Orthologs are arranged along a phylogenetic tree, with the color bar on the left indicating the mammalian orders of individual orthologs; colors are explained in the legend. For visual clarity, the phylogenetic tree was simplified, and phylogenetically estimated divergence times were omitted. Red clusters represent groups of highly similar orthologs, which align well with mammalian orders (yellow arrows), while dark blue areas indicate a lack of similarity. The three largest similarity clusters are framed, and the low sequence similarity between them is highlighted with double-sided arrows and ANI values. On the right side of the heatmap, the positions of archetypes are marked. The color code indicates the availability of RNA-seq data for the predicted gene regions in Fig. 1b and Figure S1b: red—data available, blue—data not available, yellow—human and mouse genes. (c) Bar plot of averaged ANI for specified groups of orthologs or genes, estimated in pairwise all-to-all comparisons. In addition to the two *NEAT1* isoforms, we also present *NEAT1*_3.5kb+—a part of *NEAT1Long* excluding the 5′-end of the gene, which is shared with *NEAT1Short*. Archetypes refers to a subset of 16 of the most diverged *NEAT1* orthologs. For this species subset, we estimated the average ANI of *MALAT1* orthologs and of protein-coding genes (see Methods). The averaged ANI of two structural parts of transcripts of protein-coding genes were included for comparison—CDS regions (15,461 orthologous genes were used) and 3′-UTR regions (*n* = 13,847). (d) Heatmap of primary sequence similarity among *NEAT1* archetypes. Orthologs are arranged along a phylogenetic tree, and the color bar on the left side corresponds to the mammalian orders of individual orthologs; colors are explained in the legend. The phylogenetic tree is time-scaled.

Our dataset of hundreds of *NEAT1* and *MALAT1* orthologs enabled a unique assessment of their sequence diversity across mammals and provided insight into their evolutionary patterns. In order to do this, we generated a heatmap (Fig. 4a and b) depicting the average nucleotide identity (ANI) between ortholog pairs in an all-vs-all comparison, with mammals ordered according to the phylogenetic tree. For *NEAT1*, this analysis revealed clusters of higher homology with a strong phylogenetic signal, as these clusters corresponded to mammalian orders (yellow arrows, Fig. 4b). However, between clusters, the similarity of *NEAT1* orthologs was low, in some cases barely exceeding 20% ANI (highlighted clusters, Fig. 4b). The high sequence diversity and low similarity levels limit the applicability of standard phylogenetic methods based on multiple sequence alignment (MSA), as such alignments become nearly random for the most divergent sequences.

To simplify the identification of shared gene features that may be functionally important, we selected *NEAT1* orthologs with the lowest sequence similarity to one another, which we refer to as archetypes (Fig. 4c and d). Some archetypes represented large groups of orthologs—for example, human *NEAT1* represented the cluster comprising those from Primates, Chiroptera, Carnivora, Artiodactyla, and Rodentia families other than Muridae and Cricetidae, while mouse *Neat1* served as an archetype for the Muridae and Cricetidae families (Rodentia order). The remaining archetypes originated from Monotremata, Rodentia (4 archetypes), the Lagomorpha order (2 archetypes), Marsupials (2 archetypes), Eulipotyphla (3 archetypes), Hyracoidea, and the Tenrecidae family (Afrosoricida order) (Fig. 4b and d).

Our results confirmed that *MALAT1* is much more conserved than *NEAT1*, with orthologs of Eutherians sharing 60% ANI or higher (Figure S2a, Fig. 4b) and only the orthologs of Marsupialia and Monotremata were more distinct. Overall, the clustering patterns of heatmaps for both genes were very similar, and the *MALAT1* orthologs in species encoding *NEAT1* archetypes were also among the most diverse (Figure S2b and c). Analysis of this subset of *MALAT1* orthologs revealed positions in multiple sequence alignments that were identical among the archetypes, covering approximately 13% of the *MALAT1* sequence (Figure S2d). These findings suggest a high functional importance for the primary sequence of *MALAT1*, particularly its 3′-end.

To estimate the degree of sequence variation of *NEAT1* and *MALAT1*, we compared the averaged ANI of the genes to the averaged ANI of coding sequences (CDSs) and 3′-UTRs of transcripts of orthologs of protein-coding genes in mammals (Fig. 4c). We found that *MALAT1* was nearly as conserved as CDSs, while NEAT1 exhibited conservation levels comparable to 3′-UTRs. Notably, the ANI of *NEAT1Short* was significantly higher than that of the *NEAT1_3.5kb+* region (*NEAT1Long*, downstream of 3.5 kb). The *NEAT1Short* isoform displayed some sequence similarity among archetypes, whereas similarity in the *NEAT1_3.5kb+* region was nearly absent. This is the first systematic analysis comparing the conservation level of *NEAT1Short* to the rest of the gene, with the higher conservation of *NEAT1Short* underscoring its potential functional significance.

### TEs Integrate into Specific Regions of NEAT1 and are Rarely Detected in MALAT1, Despite Nucleotide Composition

**Fig. 5.**
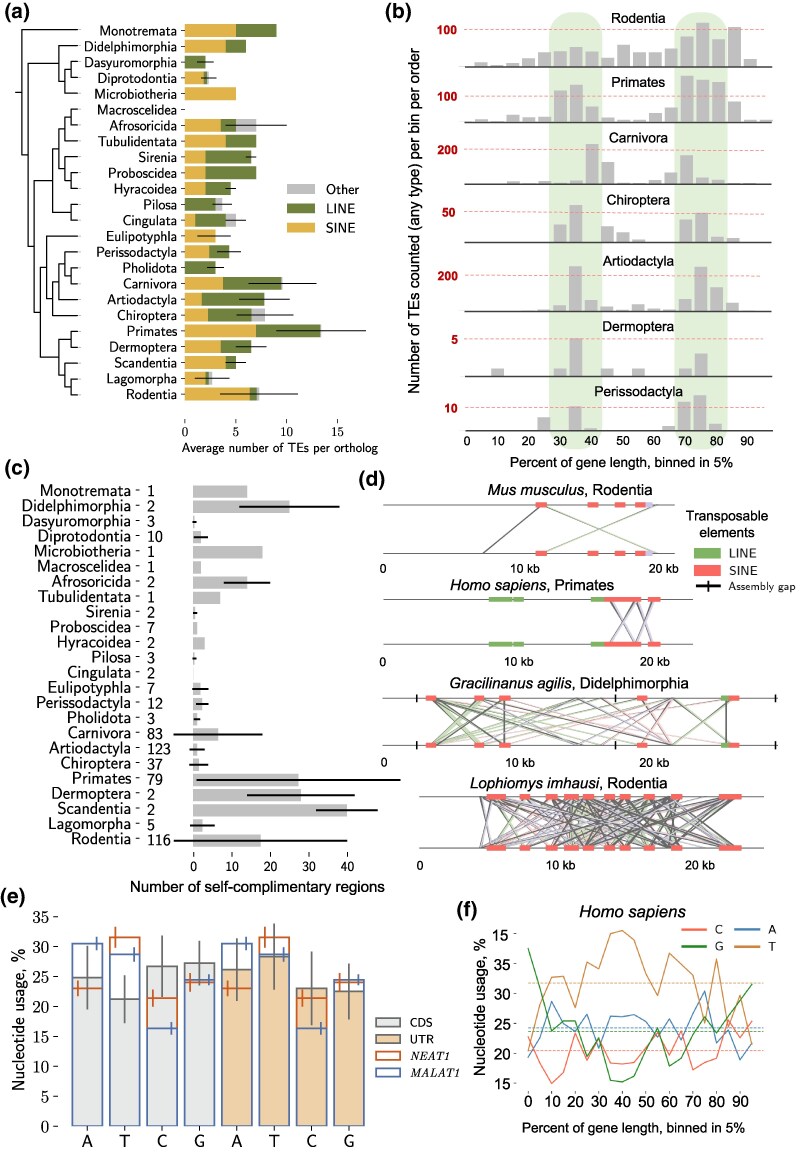
**Transposable elements contribute to *NEAT1* sequence diversity**. (a) Bar plot of the average number of TEs and their type per ortholog across mammalian orders. The phylogenetic tree is not time-scaled. (b) Distribution of TEs within *NEAT1* orthologs. The length of individual orthologs was binned into 5% segments, and the number of annotated TEs within each bin was summed for all orthologs per mammalian order. Two segments (30–40% and 70–80% bins), which most frequently contain TEs, are highlighted in green. (c) Bar plot showing the number of self-complementary regions per ortholog, averaged per mammalian order. The number of orthologs used in the assessment is indicated on the left. (d) Graphical representation of the distribution of self-complementary regions in four selected *NEAT1* orthologs. Each ortholog is aligned to itself, and reverse complementary regions are connected with lines, forming a visual “cross” shape. The second “cross” (around 20 kb) in human *NEAT1* corresponds to an IRAlu-formed hairpin. Self-complementary regions often coincide with TEs and can occur over short distances, resembling the IRAlu of human *NEAT1*, or over much longer distances. Additional plots are in Figure S3c. (e) Averaged nucleotide usage (nucleotide composition of a molecule estimated as a percentage) of *NEAT1* and *MALAT1* orthologs compared to the averaged nucleotide usage of CDS and 3′-UTRs of protein-coding genes. The nucleotide usage of *NEAT1* and *MALAT1* is overlaid on colored bars representing the nucleotide usage of CDSs (grey bars) and 3′-UTRs (orange bars). The standard deviation whisker is shifted for visual clarity. (f) Plot of nucleotide usage of human *NEAT1* along the sequence. The length of the ortholog was binned into 5% segments, and the nucleotide usage of each bin was estimated. The average nucleotide usage of human *NEAT1* is depicted with dashed lines. Additional plots for *NEAT1* are in Figure S6, and for *MALAT1* in Figure S7.

Due to the high diversity of the primary sequences of *NEAT1* orthologs, we focused on identifying shared features that could be detected without the use of MSA. We began with the analysis of TEs, which were detected in high numbers in human and mouse *NEAT1* orthologs previously (Vlachogiannis et al. 2021), and found their high diversity and enrichment in almost all *NEAT1* orthologs (Fig. 5a). We also observed that the distribution of TEs along *NEAT1* archetypes was predominantly species-specific (Figure S3b). While our TE identification method depends on how well TEs are studied in specific groups of mammals—which may affect the finding of exact TE types and frequencies—we can still gain a general impression of the importance of TEs in the evolution of *NEAT1*.

Next, we analysed the integration positions of TEs in *NEAT1* orthologs by binning the orthologs into 5% length intervals and counting the number of TEs in each bin (Fig. 5b). Summing the data per taxon, we found that a few taxa exhibited a bimodal distribution of integration sites, around 30–40% and 70–80% of the gene length. These taxa included Carnivora, Artiodactyla, Primates, and Chiroptera orders. However, in Rodentia, TEs were broadly distributed, with a slight preference for the end of the gene (Fig. 5b). Although *NEAT1* is known to be enriched in TEs, this is the first indication that it contains two predominant regions permissive to TE integration without disrupting function.

Regions of self-complementarity can potentially contribute to *NEAT1's* secondary structure formation and paraspeckle stabilization, however, have not been a focus of previous research. For example, IRAlu elements (SINE) of 3′-end of human *NEAT1*, which are regions of self-complementarity in close proximity, can form stem loops that contribute to *NEAT1* A-to-I modification and paraspeckle assembly via interaction with NONO and SFPQ (Knott et al. 2016; Vlachogiannis et al. 2021). Therefore, we studied the presence of self-complementary regions in *NEAT1* and *MALAT1* in the whole diversity of mammalian orthologs and found that these regions were common in *NEAT1* but not in *MALAT1* (Fig. 5c and d, Figure S3c). Specifically, we identified self-complementary regions in 71% of *NEAT1* orthologs, with 14.68 ± 20.85 regions per ortholog, and *Lophiomys imhausi* (Rodentia) exhibiting the maximum recorded number of 132 regions (Fig. 5d). We observed that some of these possible interactions occurred over long distances, while others were in close proximity, potentially resembling the function of IRAlu elements in human *NEAT1* (Vlachogiannis et al. 2021, Fig. 5d, Figure S3c). Additionally, the self-complementary interactions exhibited taxa-specific pattern highlighting potential evolutionary adaptations in certain mammalian groups (Fig. 5c). This diversity of interactions could be explained by the bimodal pattern of TE distribution, as we also noted that TEs were frequently the sources of these complementary regions. Overall, this is the first indication of the importance of the self-complementary regions associated with TE integration activity in *NEAT1* mammalian orthologs.

Importantly, TEs were rarely localized within *NEAT1Short* isoforms, highlighting their exposure to separate evolutionary pressures. We identified only 49 cases in six mammalian orders (Figure S4a). While it has been shown that mouse *Malat1* contains the SINE B2 element, we found this to be an exception, as our data revealed only 13 orthologs with a single TE (Figure S4b). Most of these TEs were found in Rodentia and they were localized in close proximity to the 5′-end (Figure S4b). Our original findings further highlighted the importance of *MALAT1*'s primary sequence for its function, and systematically showed that it is rarely affected by TE activity.

As SINEs typically integrate into A-T enriched regions (Daniels and Deininger 1985), we analysed nucleotide usage in *NEAT1* and *MALAT1* orthologs to gain mechanistic insight (Figure S5). We found a high enrichment of T and a depletion of C nucleotides in almost all orthologs of both genes. *MALAT1* orthologs additionally exhibited a high proportion of A nucleotides, demonstrating a nucleotide composition potentially more prone to TE integration (Fig. 5e, Figure S5). To determine how these nucleotide proportions relate to other genes, we compared them to CDS and 3′-UTR regions of protein-coding genes in mammals (Fig. 5e). This analysis showed enrichment of C and G nucleotides in CDSs and A and T nucleotides in 3′-UTRs. Additionally, it has been shown that 3′-UTRs are also prone to TE integration (van de Lagemaat et al. 2003), which aligns well with the nucleotide usage profile which we analysed. We found that *NEAT1* and *MALAT1* had similar composition to 3′-UTRs (genes were within the standard deviation), although *MALAT1* exhibited an even stronger depletion of C nucleotides. Therefore, our analysis uniquely demonstrated that from a sequence composition perspective, *MALAT1* exhibited an exceptionally low TE frequency.

Finally, we analysed nucleotide usage along the sequences of the two genes. We identified peaks of G nucleotide usage at both ends of the *NEAT1* gene, with a more pronounced peak at the 5′-end (Fig. 5f). This pattern was noticeable in almost all archetypes (Figure S6). Overall, the A-T enriched central region of *NEAT1* coincided well with the hot spots of TE integration. In *MALAT1* orthologs, the nucleotide usage pattern differed, showing a peak of A nucleotide usage at the 5′-end of the gene, which correlates with the integration sites of the infrequently detected TEs (Figure S7). In summary, we demonstrated the positional specificity of the high frequency of TE's integration in *NEAT1Long*, which corresponds well to A-T nucleotides enrichment and the presence of self-complementary interactions. In contrast, TE integration was exceptionally low in *NEAT1Short* isoforms and in *MALAT1* orthologs.

### G-quadruplexes and Binding Sites for TDP-43 are Common Features in Archetypes

The next group of features we analysed were short primary sequence patterns. Guanine tracks separated by loops can form G-quadruplexes—secondary structures which, in human *NEAT1* and *MALAT1*, facilitate interactions with NONO (Arun et al. 2020; Mou et al. 2022). We explored the universality of these structures in *NEAT1* and *MALAT1* orthologs beyond humans and predicted them in high numbers in *NEAT1* (19.2 ± 5.9 per ortholog) and *MALAT1* (9.1 ± 1.6 per ortholog).

**Fig. 6.**
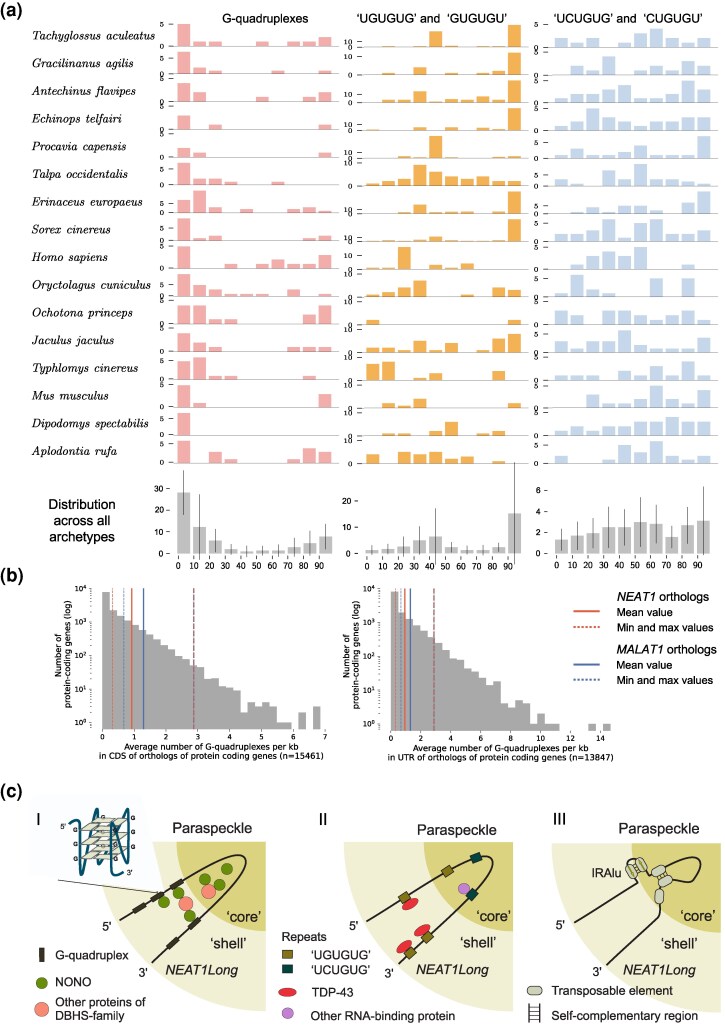
**Simple and complex repeats in *NEAT1* and *MALAT1* orthologs.** (a) Distribution of G-quadruplexes and two groups of hexamers in *NEAT1* archetypes. Each ortholog's length was divided into 10% bins, and the number of detected elements was summed per bin. In the bottom part of the panel the distribution of the studied elements is summarized for all the archetypes (mean ± std). (b) Frequency of G-quadruplex detection in CDSs and 3′-UTRs of protein-coding transcripts. The number of detected G-quadruplexes per kb in orthologs was averaged per gene and used in the plot. (c) Summary of identified conserved features of *NEAT1*, potentially contributing to the function and stabilization of paraspeckles. I. Distribution and interactions of G-quadruplexes with NONO and potentially other proteins of the DSHS family. The structure of a G-quadruplex is depicted in the zoom-in insert. II. The predominant distribution pattern of two universal hexamer sequence motifs potentially recognized by TDP-43 and other RNA-binding protein(s). III. Bimodal pattern of TEs integration, frequently associated with self-complementary interactions in close proximity, potentially forming IRAlu-like structures possibly recognized by DSHS family proteins, and also at long-range distances, possibly facilitating *NEAT1* conformation and paraspeckle stabilization.

In the *NEAT1* archetypes, they predominantly localized at both ends, within the “shell” area of the paraspeckles (Fig. 6a). This observation aligns well with our finding of nucleotide usage at both ends of *NEAT1*, showing enrichment in G nucleotides. We compared frequencies of G-quadruplexes to CDSs and 3′-UTRs of orthologs of protein-coding genes in mammals (Fig. 6b), with length-normalization applied. *NEAT1* and *MALAT1* orthologs contained more G-quadruplexes than most transcripts’ parts, especially in some individual orthologs. Our findings point to the significant importance of G-quadruplexes in both genes.

Next, we used *NEAT1* archetypes to identify frequent or systematically recurring sequence motifs that are universally important for potential paraspeckle formation and function. We chose hexamers as an optimum between diversity and uniqueness, given that the 4,096 possible combinations of letters in hexamers are theoretically diverse enough to appear only once or twice in the longest *NEAT1* ortholog, which contains 6,075 hexamers. Longer motifs are more diverse (16,384 combinations of 7-mers), making it less likely to find the same motif in all orthologs.

As a result of hexamer profiling, we identified two groups of motifs that are both frequent and common to all *NEAT1* archetypes. The first group comprised “GU”-based hexamers (“GUGUGU” and “UGUGUG”), which are known TDP-43 binding sites (Rot et al. 2017; Modic et al. 2019). These hexamers displayed largely ortholog-specific distribution patterns, with some showing a preference for the 3′-end in certain archetypes (Fig. 6a, Figure S8a). TDP-43, known to localize to the “shell” region of paraspeckles (West et al. 2016), may bind these motifs. The second group of motifs included “UCUGUG” and “CUGUGU” and was found at higher frequency and lower variability in the central region of *NEAT1*, corresponding to the paraspeckle “core”. While these motifs may also be recognized by TDP-43 (Rot et al. 2017), the difference in distribution patterns suggests distinct regulatory mechanisms and possibly varying binding affinities for TDP-43. Additionally, these motifs can be recognized by other RNA-binding proteins. We noticed that some of the identified hexamers and G-quadruplexes were located within TEs (Figure S8a), emphasizing the special role of TEs in shaping *NEAT1*'s biology. Both groups of hexamers, as well as G-quadruplexes, were also observed in non-mammalian *NEAT1* orthologs (Figure S8b, Weghorst et al. 2024), though with greater variability in distribution and abundance.

We summarized the key features of *NEAT1* sequences that are potentially important for paraspeckle function in Fig. 6c. This underscores the importance of G-quadruplexes, TDP-43 binding motifs, and self-complementary regions, which can potentially determine the functional interactions with proteins essential for paraspeckle assembly, even in the absence of primary sequence conservation.

### Taxa-specific Speed of *NEAT1* Evolution

**Fig. 7.**
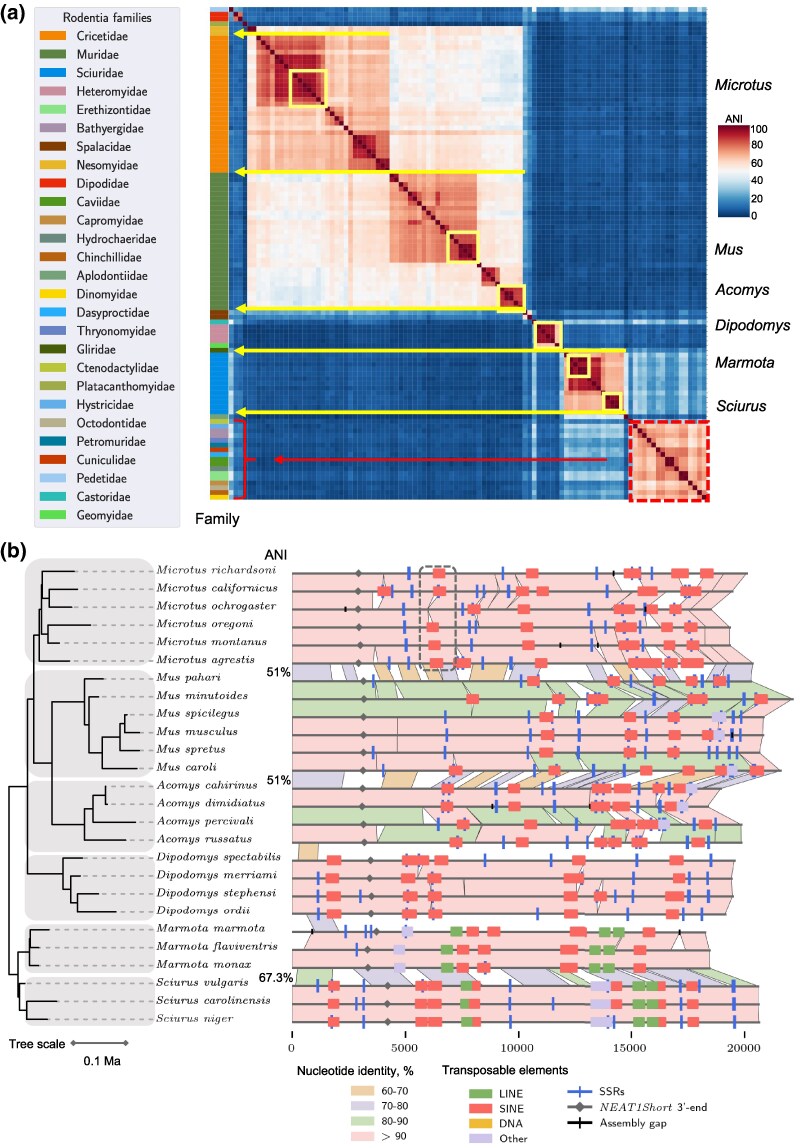
**Uneven speed of *NEAT1* evolution.** (a) Heatmap of primary sequence similarity between *NEAT1* orthologs of the Rodentia order, in pairwise all-to-all comparison. Clusters of red represent groups of highly similar orthologs which aligns well to family borders (yellow arrows), while dark blue areas indicate a lack of similarity between them. The color bar on the left represents individual families, as seen in the legend. Six selected genera for the alignment in part B are highlighted with yellow frames. The red dashed frame highlights the similarity cluster of 13 Rodentia families mentioned in the Results section. (b) Graphical representation of the pairwise alignment of *NEAT1* orthologs from six Rodentia genera. Orthologs are aligned according to the time-scaled phylogenetic tree on the left. Individual genera are highlighted in grey for visual clarity. *Mus* and *Acomys* genera exhibit a higher evolutionary rate compared to the others. An example of an excised SINE element is highlighted with a dashed frame.

This uniquely large collection of *NEAT1* orthologs enabled us to uncover previously unrecognized patterns in its evolutionary development. The divergence of primary sequences of *NEAT1* orthologs cannot be explained solely by the phylogenetic tree and the evolutionary time since the taxa split. We observed this by examining the ANI of orthologs within mammalian orders (Fig. 4a). For example, orthologs of Carnivora or Artiodactyla are highly similar to each other (60 to 70% ANI), whereas Lagomorpha or Eulipotyphla include several archetypes with ANI lower than 10%. Another notable observation is that Rodentia and Lagomorpha are phylogenetically closer to Primates than to Carnivora, yet orthologs of Primates are much more similar to Carnivora than to those of Rodentia and Lagomorpha. We focused on the Rodentia order, as it comprised the largest number of identified orthologs and exhibited high diversity in their primary sequences (Fig. 7a and b). Within this order, we observed that different families contributed orthologs with varying levels of similarity within a taxon (e.g. Muridae and Cricetidae families). The similarity between taxa could be high for some families (red dashed cluster, Fig. 7a) and very low for others (yellow arrows, Fig. 7a). Thus, taxonomic borders within the Rodentia order also did not adequately explain the variability of *NEAT1* sequences.

Next, we sought to identify possible drivers of *NEAT1* evolution and analysed orthologs originating from six Rodentia genera for which we had at least three species (Fig. 7b). The highest primary sequence variation was detected in the genera *Mus* and *Acomys*, while other genera exhibited relatively high levels of conservation. This difference in the rate of evolution was also visible between the genera. Specifically, the evolutionary divergence of *Sciurus* and *Marmota* occurred earlier than that of *Mus*, *Acomys*, and *Microtus*, yet the similarity of orthologs between *Sciurus* and *Marmota* was the highest (67.3% ANI, Fig. 7b). Although *Mus* and *Acomys* diverged later than either of them from *Microtus*, the similarity level was the same (51% ANI) between *Mus* and *Acomys* and between *Acomys* and *Microtus*. This suggests a high rate of evolution in *Mus* and *Acomys* and a greater conservation in *Microtus*.

In the analysed graphical alignments of *NEAT1* orthologs, we noticed that the most varied regions are frequently associated with sites enriched in TEs (Fig. 7b, see also Figures S9 and S10). Our collection of orthologs included several species (e.g. *Mus musculus*), for which multiple genome assemblies existed, resulting in several *Neat1* variants (Figure S9). In these cases, sequence divergence was minimal, and only TE integration events accounted for the differences. Therefore, our results clearly demonstrate that an accelerated mutational process, accompanied by high TE integration activity, was a major driver in the evolutionary shaping of *NEAT1*, with a clear taxon-specific pattern.

One key difference between SINE and LINE elements is that SINEs depend on LINEs for amplification. Moreover, a specific mechanism for SINE excision has not been identified (Batzer and Deininger 2002), suggesting that SINEs remain at their integration site and erode through mutational process (Richardson et al. 2015). However, we identified three rare but clear examples of SINE excision. For example, a SINE shared by the entire *Microtus* genus was excised in *Microtus ochrogaster* (highlighted area, Fig. 7a, Figure S10). This observation suggests the existence of a mechanism for SINE excision and indicates that, overall, TE dynamics is one of the major factors shaping *NEAT1* evolution.

## Discussion

*NEAT1* is a paradoxical lncRNA: it lacks sequence similarity between orthologs yet retains functionality, as confirmed for the four *NEAT1* archetypes of human, mouse, naked mole-rat and opossum. Here, by leveraging the extensive and diverse dataset of poorly conserved *NEAT1* orthologs, we investigated the factors contributing to its functional conservation by applying a strategy to identify smaller structural elements in phylogenetically diverse orthologs. A conserved feature of the *NEAT1* gene, as indicated by our research, is that it gives rise to three molecules—*NEAT1Long*, *NEAT1Short*, and a tRNA-like structure, which we discuss separately.

### Architectural Long *NEAT1* Isoform

*NEAT1* is one of the longest known lncRNA gene in the human genome and possibly in all mammals, as our results suggest. Functionally, longer RNAs enable faster and more efficient condensate formation, as demonstrated using synthetic RNAs (reviewed in Van Treeck and Parker 2018; Garcia-Jove Navarro et al. 2019). Similarly, studies have shown that RNAs isolated from stress granules are significantly longer than cytoplasmic RNAs which do not localize to stress granules (Khong et al. 2017). The remarkable length of *NEAT1*, which has not previously been the focus in relation to its functional impact, may also explain why paraspeckle formation begins immediately at the transcription site, with multidomain stabilizing proteins recruited later (Mao et al. 2011). The substantial variation in the length of *NEAT1* orthologs raises an open question about the potential variation in the physical properties of paraspeckles across species, such as differences in the speed of paraspeckle assembly, their linear size and stiffness.

We discovered that GU repeats are a universal feature of *NEAT1* archetypes and are frequently localized near the 3′ end. Previously, we demonstrated that the isoform switch of human and murine *NEAT1* is regulated by TDP-43 (Modic et al. 2019), which is typically localized in the “shell” region of paraspeckles (West et al. 2016). A decrease in TDP-43 availability, also caused by its sequestration into paraspeckles, prevents polyadenylation of the short isoform (Modic et al. 2019). In the same study, we also showed that GU repeats are the predominant mechanism for TDP-43 sequestration in paraspeckles. Thus, the interaction between *NEAT1* and TDP-43 is clearly a crucial functional aspect of paraspeckles, and our findings provide the first indication that the mechanism of isoform switching via TDP-43 association with GU repeats in *NEAT1* is likely conserved across all mammals.

G-quadruplexes are secondary structures common to all *NEAT1* orthologs in their 3′ and 5′ regions. Functionally, G-quadruplexes are involved in almost all aspects of gene expression regulation, from transcription to translation, in the modification of mRNAs and miRNAs, and in phase separation processes (Asamitsu and Shioda 2021; Dumas et al. 2021). Importantly, we noted that the list of RNA-binding proteins capable of interacting with G-quadruplexes overlaps with paraspeckle proteins (Fox et al. 2018; Bourdon et al. 2023). For example, paraspeckle proteins—HNRNPH3, HNRNPK, RBM14, SMARCA4, NONO, SFPQ, and TDP-43—along with 17 other non-essential proteins, including PSPC1, are all capable of binding to G-quadruplexes. The diversity of paraspeckle proteins that recognise G-quadruplexes suggests the potential for interchangeability in maintaining paraspeckle integrity, which may explain the importance but non-essentiality of certain proteins (Fox et al. 2018). Another protein that potentially binds to G-quadruplexes of *NEAT1* is SRSF1—a protein actively involved in splicing regulation, predominantly localized in nuclear speckles and regulated by *MALAT1* (Romero-Barrios et al. 2018; Yamada et al. 2022; De Silva et al. 2024). Additionally, SRSF1 binds to and stabilizes *NEAT1* RNA, which consequently affects the cell cycle (Zhou et al. 2019). Overall, G-quadruplexes provide a potential mechanism for the recruitment of mRNAs, miRNAs, and proteins to paraspeckles. We also speculate that G-quadruplexes, which are formed by DNA as well (Asamitsu and Shioda 2021; Dumas et al. 2021), may be utilized by G-quadruplex-binding proteins to cross-stitch paraspeckles to DNA.

We found that many *NEAT1* orthologs are characterized by reverse complementary regions, frequently originating from diverse TEs. In human *NEAT1*, IRAlu can form stem-loop structures that attract ADAR enzymes, which modify A-bases to-I, and are potentially bound by NONO (Elbarbary and Maquat 2015; Vlachogiannis et al. 2021). By extension, we postulate that the complementary regions, which we identified in high abundancies in many orthologs, may have the potential to form stem-loop structures and interact with NONO and/or ADAR enzymes. Another possibility is that in cases where complementary regions are interspersed, they might contribute to paraspeckle stabilization, particularly in the early phase of paraspeckle assembly before the recruitment of multidomain proteins.

### Short Isoform of *NEAT1*

Our original results highlight the universality of *NEAT1Short* and the higher conservation of its primary sequence and isoform length compared to *NEAT1Long*. We detected only a small number of cases where *NEAT1Short* contained TEs, and overall, *NEAT1Short* was depleted of both simple and more complex repeats. These findings indicate a distinct functional trajectory for *NEAT1Short*, separate from *NEAT1Long*, about which little is currently known. For example, *NEAT1Short* has recently been associated with TIRR, an RNA-binding protein that interacts directly with 53BP1, restricting its access to DNA double-strand breaks and its association with p53 (Kilgas et al. 2024). It has been shown that *NEAT1Short* can be located outside of paraspeckles and concentrated in much smaller foci known as “microspeckles,” the function of which remains unclear (Li et al. 2017). In experiments conducted by Naveed et al., it was demonstrated that *NEAT1Short* can have an effect on cell proliferation that is opposite to that of *NEAT1Long* (Naveed et al. 2021).

### tRNA-like Structure

The primary sequences of tRNA-like structures are highly conserved not only within *NEAT1* or *MALAT1* orthologs but also between the two genes. Our dataset, which significantly expands the number and diversity of known *NEAT1* and *MALAT1* sequences and their structural elements, allows for improved identification of the most conserved regions. Comparing co-evolving structures in our analysis of 545 species with those identified in a smaller dataset (Marz et al. 2014) highlights the broader diversity of tRNA-like primary sequences within mammals. This higher sequence diversity, in turn, helps pinpoint the most functionally important structural components—specifically, the highly conserved hairpin III (Fig. 2a and b) and the overall tRNA-like conformation, which are likely key elements in the maturation processes of both *NEAT1* and *MALAT1*.

Differences in the conservation levels of tRNA-like structures in *NEAT1* and *MALAT1* orthologs may indicate functional divergence. It has been shown that *MALAT1*'s mascRNA may additionally play a role in cellular metabolism within the cytoplasm. For example, it can contribute to increased protein translation and cell proliferation by binding to the multi-tRNA synthetase complex (Lu et al. 2020). Dissimilarly, *NEAT1*'s tRNA-like molecules were shown to degraded in human cell lines (Wilusz et al. 2008, 2011). Based on these differences, it is important to systematically analyse the functions of tRNA-like molecules in different cell types and animals, as they may have been adapted for specific functions.

### From Conserved Transcriptional Regulation to *NEAT1*'s Role in Cell Biogenesis

The identification of TF motifs shared by hundreds of mammalian species in the *NEAT1* and *MALAT1* promoters suggests their involvement in specific cellular and molecular pathways. Although our study presents the first large-scale computational prediction of potential biological processes for both genes, we observed a strong concordance between our results and previously reported experimental findings. For example, *NEAT1* has been implicated in apoptosis and proliferation (Adriaens et al. 2016; Kilgas et al. 2024), as well as in diverse neurodegenerative diseases (An et al. 2018), potentially via the same TFs involved in CNS development. The number of paraspeckles (and *NEAT1* expression levels) oscillates with circadian rhythms, releasing IRAlu-containing mRNAs (Torres et al. 2016, 2017) and regulating 53BP1 availability in a cell-cycle-dependent manner (Kilgas et al. 2024). Moreover, *NEAT1* directly binds approximately 30% of all mRNAs located in paraspeckles, most of which are also involved in circadian rhythm cycles (Jacq et al. 2021). Additionally, NONO and SFPQ are known to be involved in circadian rhythm regulation (Kowalska et al. 2012; Knott et al. 2016). Our analysis also suggests a potential role for *NEAT1* and *MALAT1* in spermatogenesis and gonad development, which aligns well with the findings of Zhang et al., demonstrating that many *MALAT1*-like genes in *Anolis carolinensis* are highly expressed in the testis and enriched in the nuclei of round spermatocytes (Zhang et al. 2017).

### *NEAT1* and *MALAT1*: Uniquely Similar but Different lncRNAs

Our study confirms the synteny of *NEAT1* and *MALAT1* across the full range of mammalian species. The uniqueness and similarity of their gene maturation processes along with their roles in spatially associated nuclear bodies, raise the expectation of similar regulation, conservation, and function for *NEAT1* and *MALAT1*. However, this is not the case: *MALAT1* is a highly conserved lncRNA, while *NEAT1* is more variable.

This difference in conservation is possibly associated with the frequency of TEs integration, as *NEAT1* is more prone to such integrations compared to *MALAT1*. However, our analysis of nucleotide usage highlighted an opposite trend: *MALAT1* has, on average, a more favorable nucleotide composition for TE integration. This further underscores the functional importance of conserved primary sequence of *MALAT1*. It has been shown that two regions in *MALAT1*, located approximately at 2 to 3 kb and 6 to 7 kb, are responsible for its localization in nuclear speckles (Miyagawa et al. 2012), which aligns with our results showing a high level of sequence conservation in these regions. The accumulation of mutations in another conserved region of *MALAT1* (3 to 4.3 kb) has been associated with breast cancer progression (Ellis et al. 2012), highlighting the importance of an intact primary sequence for proper function under normal physiological conditions. Together, these findings suggest that *MALAT1*'s primary sequence plays a major role in its function, while for *NEAT1*, secondary structural elements appear to be more crucial.

The analysis of the conservation of promoter regions, TATA-boxes, and transcription TF binding sites revealed another key difference between *NEAT1* and *MALAT1*. Although *MALAT1* showed greater gene conservation than *NEAT1*, the variability in *MALAT1*'s promoter region and potential transcriptional regulation was higher. This provides an indication that *MALAT1* may have adapted to different gene networks across species, while *NEAT1* remains a consistent player in the same biological processes.

### Uneven Speed of *NEAT1* Evolution

Our research identified two main mechanisms driving *NEAT1* evolution: divergence due to the accumulation of mutations and the high frequency of TEs integration and excision. It is widely accepted that TEs play a significant role in mammalian evolution (Senft and Macfarlan 2021). Intergenic lncRNAs are much more enriched in TEs compared to protein-coding genes (Hezroni et al. 2015) and the most common TE type in lncRNAs is ERVs, while SINEs and LINEs are depleted (Kelley and Rinn 2012). *NEAT1* is known to be enriched in repeats (Souquere et al. 2010) and here we demonstrate both the diversity and the impact of TEs on *NEAT1* evolution.

TEs influence gene length in both directions—making it longer through integration or shorter through excision—explaining the considerable variation in gene length across mammals. TEs also introduce self-complementary regions, stabilizing paraspeckles, as well as repeats and G-quadruplexes, which serve as interaction sites for key resident proteins. This observation highlights the benefits of TEs integration for *NEAT1* function within paraspeckles. However, the bimodal pattern of TEs integration hot spots supports the idea that *NEAT1* cannot tolerate insertions throughout its sequence—particularly not within the 5′-end shared with the *NEAT1Short* isoform. Therefore, TEs play a crucial role in *NEAT1* evolution overall.

The consequences of TE integration into lncRNAs are variable, and *NEAT1* is not unique in being shaped by TEs. For example, TE insertions in the *ANRIL* lncRNA have been linked to increased gene conservation in primates (He et al. 2013). TEs also support *ANRIL*'s function in the trans-activation of a range of target genes, some of which are contributing to coronary artery disease (Holdt et al. 2013; Alfeghaly et al. 2021). *XIST*, another lncRNA enriched in TEs, provides further evidence of functional adaptation—where TEs have contributed to the formation of specific exons (Elisaphenko et al. 2008).

Our analysis highlighted taxa with accelerated *NEAT1* evolution, such as Eulipotyphla, Lagomorpha, and the *Mus* and *Acomys* genera of the Rodentia order. This phenomenon of varied evolutionary speed has been previously demonstrated for some lncRNAs. For example, unannotated and largely non-coding human accelerated regions (Pollard et al. 2006a, 2006b) are conserved genomic regions across mammals that accumulate disproportionately more mutations in humans, many of which function as enhancers in neurodevelopment (Doan et al. 2016; Girskis et al. 2021). Although signs of positive selection in local secondary structures of human *NEAT1* have been reported (Walter Costa et al. 2019), our data do not support the hypothesis of accelerated evolution of *NEAT1* in the human lineage. The rate of evolution highlights species or taxon-specific adaptations to their ecological niches. We speculate that this mechanism may also influence *NEAT1* biogenesis, as *NEAT1* can directly interact with diverse mRNAs and miRNAs, possibly via complementary interactions of primary sequences. This may explain the high evolutionary speed observed in certain taxa and across mammals in general.

## Material and Methods

### Identification of Coordinates for *NEAT1* and *MALAT1* Orthologs

Mammalian genomes were downloaded from GenBank (Clark et al. 2016, July 2023). Annotated *NEAT1* and *MALAT1* orthologs from *Homo sapiens* (NR_131012.1), *Mus musculus* (NR_131212.1, O’Leary et al. 2016), and *Monodelphis domestica* (KX036207.1, Cornelis et al. 2016) were used for similarity searches and the identification of orthologs in the downloaded genomes. We additionally retrieved promoter regions and triple helix motifs, followed by tRNA-like structure sequences, for these annotated orthologs using in-house scripts. These sequences were subjected to a blastn (Altschul et al. 1990) search against the downloaded mammalian genomes. Approximate gene coordinates were obtained from the homology search results and were complemented with some manual curation in cases where *NEAT1* and *MALAT1* orthologs were found on different contigs due to fragmentary assembly. Genes were retrieved with some sequence excess at both the 5′- and 3′-ends and subjected to MSA (MAFFT, v7.487, Katoh and Standley 2013), default parameters). Since *NEAT1* showed noticeably higher divergence compared to *MALAT1*, we divided the mammals into eight groups according to the phylogenetic tree (Upham et al. 2019).

Group1: Monotremata, Didelphimorphia, Microbiotheria, Diprotodontia, Dasyuromorphia

Group2: Eulipotyphla, Perissodactyla, Pholidota

Group3: Macroscelidea, Pilosa, Proboscidea, Afrosoricida, Cingulata, Sirenia, Tubulidentata, Hyracoidea

Group 4: Lagomorpha, Rodentia, Scandentia

Group 5: Primates, Dermoptera

Group 6: Artiodactyla

Group 7: Chiroptera

Group 8: Carnivora

We then added the most relevant, phylogenetically closest annotated *NEAT1* ortholog(s) to these groups and performed MSA. MSA was visualized using the online tool AlignmentViewer (https://alignmentviewer.org/). The coordinates of the genes' start and stop sites (TATA-box and end of the triple helix) within the MSA were identified and used to build the final set of orthologs and their structural elements. The same procedure was applied for the *MALAT1* ortholog search, but sequences were divided into two groups: the aforementioned Group 1 and the remaining sequences.

Subsequently, we manually curated the results and removed orthologs with excessive assembly gaps or spurious sequences lacking the correct start or end. Coordinates, contig accessions, genome assembly versions, and other results and metadata can be found in Table S1.

We examined the strand and genomic distance between *NEAT1* and *MALAT1*. Out of 428 organisms in which both genes were predicted, 92% had these genes located on the same contig. Since not all species possess complete chromosome-level assemblies, some genes were found on different contigs. In such cases, it is not possible to determine the true genomic positions of the genes. Among those located on the same contig, only two species had *NEAT1* and *MALAT1* coded on opposite strands. Assemblies of both these species, *Rousettus madagascariensis* and *Oryctolagus cuniculus*, do not belong to the GenBank reference set. After manual inspection, we found another reference assembly for *Oryctolagus cuniculus* (Figure S1b) and checked the strand and location of the predicted orthologs of *NEAT1* and *MALAT1*. Although these orthologs showed high sequence similarity to the reference assembly, the directionality of the genes was different: they were coded on the same strand, consistent with the majority of other orthologs. However, we cannot assess the impact of assembly quality on the opposing directionality observed in *Rousettus madagascariensis*, as no reference assembly is currently available.

### Comparison of *NEAT1* and *MALAT1* Orthologs to the Results of Yamada *et al* and Weghorst *et al*

Our collection included a newer version of the naked mole-rat genome assembly than the one used in the publication by Yamada et al. (Yamada et al. 2022). We downloaded the assembly used in that study and performed a blastn search of the *Neat1* sequence identified in our study against this assembly. Our start coordinate for *Neat1* was 20,972,753, which is 204 bp downstream of the start coordinate reported by Yamada et al. (JH602080:20,972,549, with both genes coded on the minus strand). The start coordinate for *Malat1* was 20,907,466, which is 60 bp downstream of the coordinate reported by Yamada et al. (JH602080:20,907,406). We assume that the 3′-ends of the genes in the naked mole-rat are identical to those we identified, as Yamada et al. also defined them computationally based on the similarity of triple helix and tRNA-like motifs.

The higher agreement we found for the koala *Neat1* (Weghorst et al. 2024), where the starting coordinate differed by 12 bp only and the 3′-end was the same. The coordinates for koala *Malat1* were identical.

### Prediction of Short Isoform in *NEAT1* Orthologs

We divided the orthologs into two similarity groups, with marsupials and Monotremata (Group 1) sequences placed separately. The remaining orthologs were subjected to MSA (MAFFT, default parameters). We identified the position in the MSA corresponding to the PAS of human *NEAT1Short* and searched for the predicted PAS in the vicinity of this position in the orthologs. PAS were predicted by searching for the canonical motif “AATAAA”. If a single signal was detected within 110 bp (in both directions) of the PAS position in human *NEAT1*, it was considered an active PAS for the *NEAT1Short* orthologs.

In Group 1, we searched for two PASs using the same logic, based on the predicted sites for *Monodelphis domestica* (Cornelis et al. 2016). In this prediction, there were three orthologs where we could not identify a single alternative polyadenylation signal, and these were omitted from the analysis.

### Prediction of TEs in *NEAT1* and *MALAT1* Orthologs

We used the DFAM database (downloaded in April 2022, Storer et al. 2021) of TEs and searched for similarities using blastn algorithm and applying the 80-80-80 rule (a minimum alignment length of 80 bp with 80% nucleotide identity over an alignment covering at least 80% of the TE). Only non-overlapping TE annotations were selected.

Using this method, we identified four large fragments of LINE elements and six complete SINEs in human *NEAT1*: four *Alu* elements and two FLAM-C elements. Two of the identified *Alu* elements, *AluSx3* (17,804 to 18,067 bp) and *AluJr* (17,532 to 17,678 bp), can form an IRAlu secondary stem-loop structure, which may attract ADARs for A-to-I modification of *NEAT1* (Vlachogiannis et al. 2021). In mouse *NEAT1*, we identified four SINE elements (*B1_Mus1*, *B3*, *B1_Mm*, and *B1_Mus2*), which are non-complementary to each other.

### Prediction of Sequence Elements in *NEAT1* and *MALAT1* Orthologs

We retrieved 1 kb of promoter sequence for each ortholog of *NEAT1* and *MALAT1* and predicted transcription factors binding sites using FIMO tool (Grant et al. 2011, part of MEME package v5.0.5, Bailey et al. 2015) and JASPAR database (core part, version 2022, vertebrates, Rauluseviciute et al. 2024). Sites with *P*-value < 10^−4^ were considered. GO terms were downloaded in October 2021 (Ashburner et al. 2000; The Gene Ontology Consortium et al. 2023), each gene was associated with all connected to it terms.

G-quadruplexes were predicted using pqsfinder R package (v.2.2.0, Hon et al. 2017).

Kmers were counted using in-house script.

Self-complementary regions were assessed from the blastn search against an ortholog itself, and only reverse complementary hits were counted.

### ANI Calculation

ANI between two sequences was calculated by using all blastn hits longer than 100 bp and following the formula:


$$ANI=\left(\frac{\sum (blastn\;hits\;Gene1)}{Length\;Gene1}+\;\frac{\sum (blastn\;hits\;Gene2)}{Length\;Gene2}\right)/2*100$$

where


$$blastn\;hit=\frac{blast\;pident}{100}*Length\;blast\;HSP$$

### Analysis of CDS and UTR Regions of Protein-coding Gene Orthologs in Mammals

CDS and UTR regions of protein-coding gene orthologs in mammals were retrieved from GenBank (Clark et al. 2016) in September 2024 using the NCBI Datasets tool (O’Leary et al. 2024, command: *datasets download gene symbol “$GENE” –ortholog mammals –include gene,cds,3p-utr,product-report*; for genes with at least 150 orthologs from different genera of our collection). In cases where multiple transcripts were available, the longest single transcript per ortholog was selected. ANI, G-quadruplexes, and nucleotide usage were predicted in the same manner as for *NEAT1* and *MALAT1* orthologs; values were averaged across all orthologs per gene before being used in distribution plots. A total of 15,461 protein-coding genes were included in the CDS analysis, and 13,847 genes were used in the UTR analysis.

### Phylogenetic Tree

The phylogenetic tree of Upham et al. (Upham et al. 2019) was used. Species for which *NEAT1* and *MALAT1* orthologs were identified but absent in the phylogenetic tree were associated with their closest relatives. A full list of these connections can be found in the Part3_PhylogeneticTree python notebook (https://github.com/kseniaarkhipova/NEAT1-MALAT1). Visualization and graphical adjustments of the phylogenetic tree were made using the iTOL web-server (Letunic and Bork 2024). Tree parsing, pruning, and the retrieval of time information were performed using the ete3 Python package (v.3.1.2, Huerta-Cepas et al. 2016).

### Other Used Resources and Software

RNAfold web-server (Lorenz et al. 2011) was used to predict and visualise folding of structural elements. LocRNA software (v. 2.0.0, http://rna.informatik.uni-freiburg.de, Will et al. 2012) was used to analyse coevolutionary patterns of tRNA-like structures. Sequence logos were generated using WebLogo web-server (Crooks et al. 2004). Taxonomic tree of NCBI (downloaded on April 2022, Schoch et al. 2020) was used to classify the studied genomes. Most of analysis was performed with customs scripts, which were written in Python 3.7.0 and used the following packages: scipy (v.1.7.1, Virtanen et al. 2020), numpy (v. 1.18.5, Harris et al. 2020), pandas (v 1.1.5, https://zenodo.org/records/10957263), matplotlib (v.3.4.3, Hunter 2007), seaborn (v. 0.11.2, Waskom 2021) and Jupyter notebook (v.4.8.1, Kluyver et al. 2016). Code and orthologs sequences are available on GitHub (https://github.com/kseniaarkhipova/NEAT1-MALAT1, DOI:0.5281/zenodo.15147921).

## Supplementary Material

msaf265_Supplementary_Data

## Data Availability

Code and orthologs sequences are available on GitHub (https://github.com/kseniaarkhipova/NEAT1-MALAT1, DOI:0.5281/zenodo.15147921).
