# Supplementary material for: Phylogenetic Analysis of NEAT1 and MALAT1 Long Non-Coding RNAs Highlights Structure–Function Relationships in Paraspeckle Biology
Source: Mol Biol Evol. 2025 Dec 9;43(2):msaf265. doi: 10.1093/molbev/msaf265 (PMC12941219; doi:10.1093/molbev/msaf265)
Supplement: msaf265_Supplementary_Data [file msaf265_supplementary_data.zip › SupplementaryFigures.pdf]

Supplementary Figure 1. Identification of *NEAT1* and *MALAT1* orthologs in mammals.
A. Number of identified orthologs in different mammalian orders. Species in which *NEAT1* or *MALAT1* orthologs were not found are highlighted in red.
B. Transcription in regions with predicted *NEAT1* and *MALAT1* orthologs for species coding *NEAT1* archetypes. Predicted coordinates were overlaid on mapped transcriptomic read profiles in the Genome Browser (*http://genome.ucsc.edu)*. In the 'Genes' section of the Genome Browser, the automatically predicted genes identified in the region are shown.
C. Distribution of the genomic distance between *NEAT1* and *MALAT1* orthologs in studied mammals.

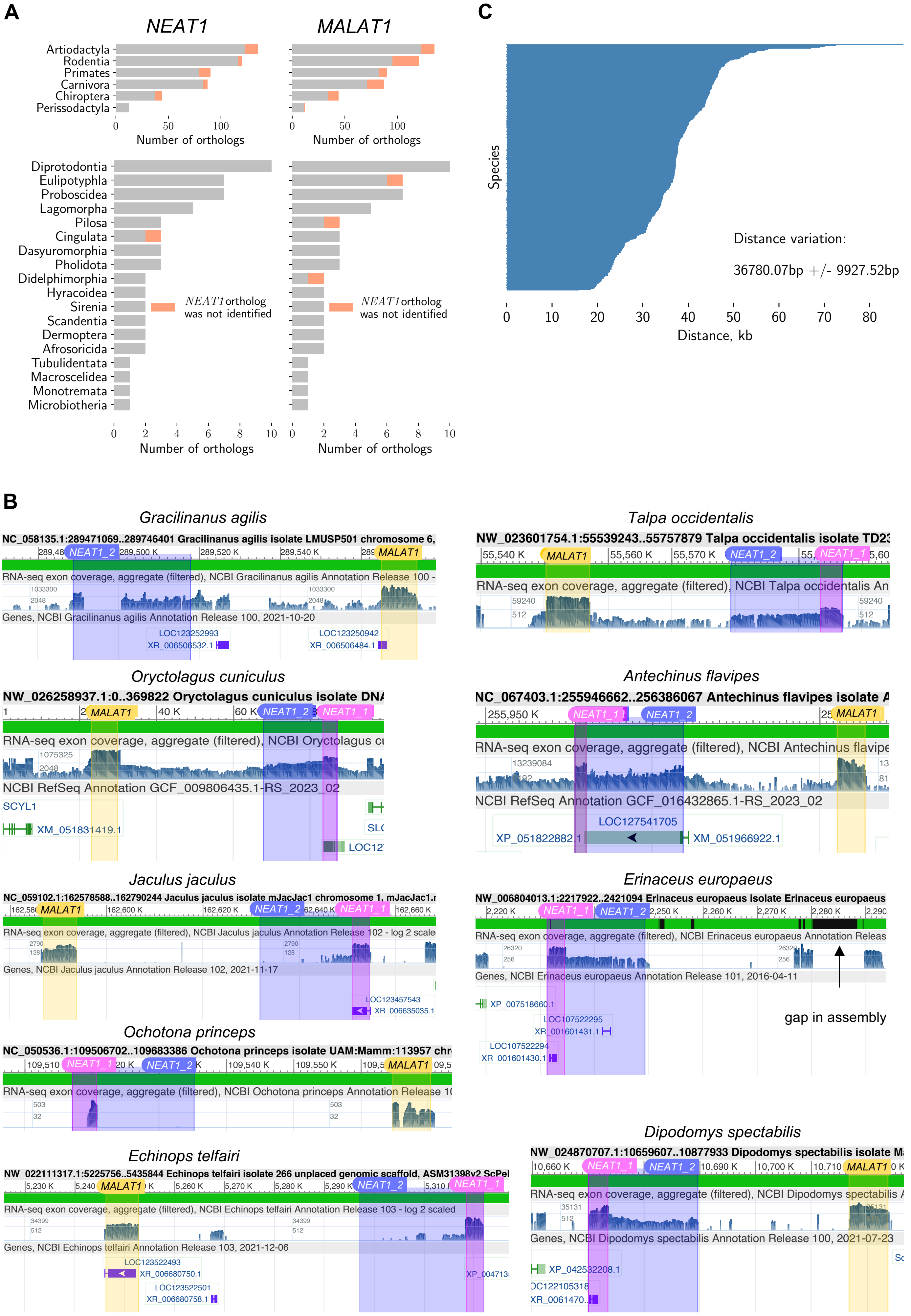
A
NEAT1
MALAT1
Artiodactyla
Rodentia
Primates
Carnivora
Chiroptera
Perissodactyla
Number of orthologs
Diprotodontia
Eulipotyphla
Proboscidea
Lagomorpha
Pilosa
Cingulata
Dasyuromorphia
Pholidota
Didelphimorphia
Hyracoidea
Sirenia
Scandentia
Dermoptera
Afrosoricida
Tubulidentata
Macroscelidea
Monotremata
Microbiotheria
NEAT1 ortholog was not identified
Number of orthologs
C
Species
Distance variation:
36780.07bp +/- 9927.52bp
Distance, kb
B
Gracilinanus agilis
Talpa occidentalis
Oryctolagus cuniculus
Antechinus flavipes
Jaculus jaculus
Erinaceus europaeus
gap in assembly
Ochotona princeps
Dipodomys spectabilis
Echinops telfairi
NEAT1_1
NEAT1_2
MALAT1

Supplementary Figure 2. Primary sequence diversity of *MALAT1* orthologs in all mammals and the subset of archetypes.

A. Heatmap of ortholog similarity in pairwise comparisons (all-to-all). Orthologs are arranged along a phylogenetic tree, and the colour bar on the left corresponds to the mammalian orders of individual orthologs; colours are explained in the legend. For visual clarity, the phylogenetic tree was simplified, and information about phylogenetically estimated time was omitted. Red clusters represent groups of highly similar orthologs, while blue areas indicate lower similarity between them.

B. Heatmap of primary sequence similarity among *MALAT1* orthologs coding *NEAT1* archetypes. Orthologs are arranged along a phylogenetic tree, and the colour bar on the left corresponds to the mammalian orders of individual orthologs; colours are explained in the legend. The phylogenetic tree is time-scaled. *MALAT1* orthologs in the subset are also among the most diverged.

C. Graphical representation of pairwise alignment between *MALAT1* archetypes. Patches of similarity longer than 100bp are depicted.

D. Regions of identical primary sequence in *MALAT1* orthologs from the archetypes subset. Regions are mapped onto human *MALAT1*.

ANI = averaged nucleotide identity.

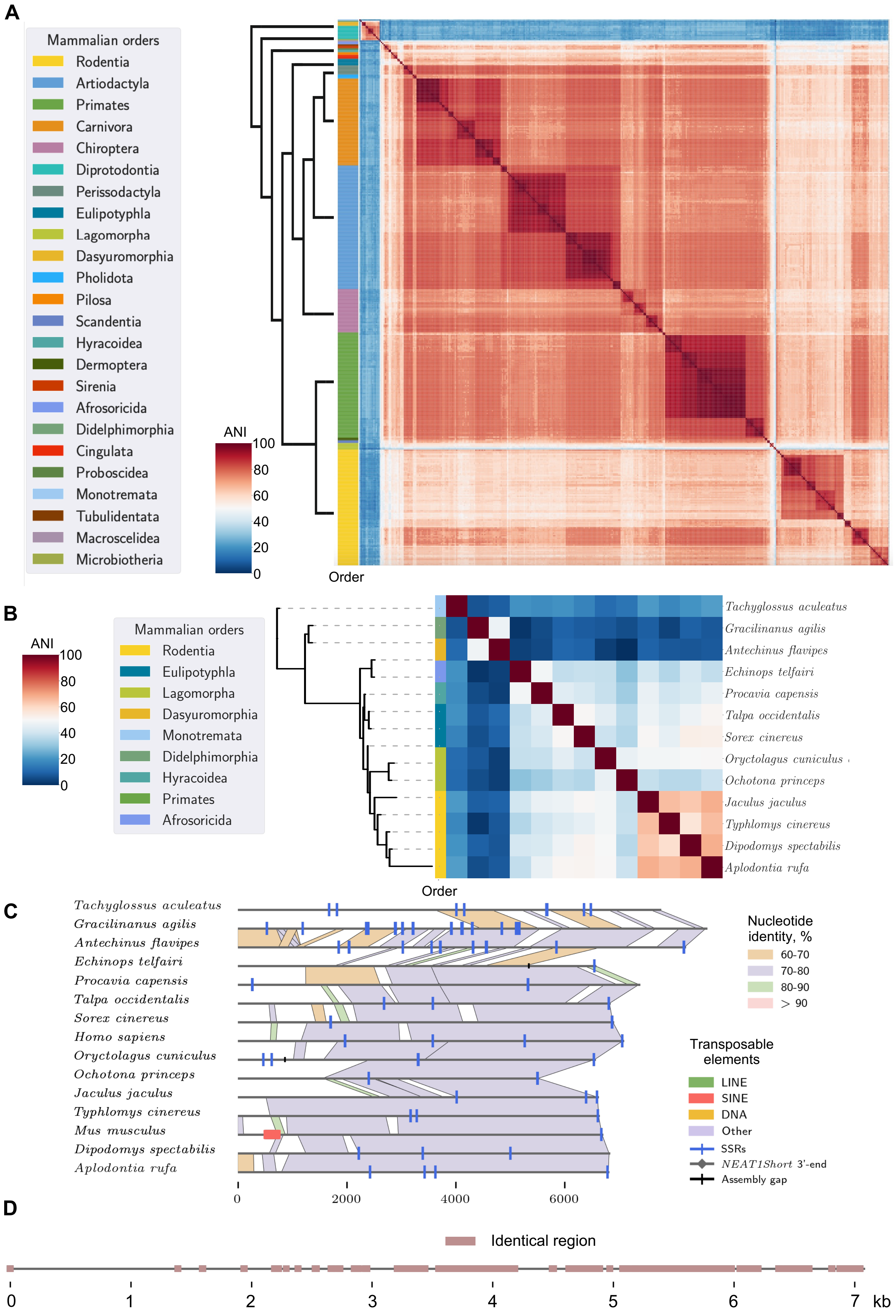
A
Mammalian orders
Rodentia
Artiodactyla
Primates
Carnivora
Chiroptera
Diprotodontia
Perissodactyla
Eulipotyphla
Lagomorpha
Dasyuromorphia
Pholidota
Pilosa
Scandentia
Hyracoidea
Dermoptera
Sirenia
Afrosoricida
Didelphimorphia
Cingulata
Proboscidea
Monotremata
Tubulidentata
Macroscelidea
Microbiotheria
ANI
100
80
60
40
20
0
Order
B
ANI
100
80
60
40
20
0
Mammalian orders
Rodentia
Eulipotyphla
Lagomorpha
Dasyuromorphia
Monotremata
Didelphimorphia
Hyracoidea
Primates
Afrosoricida
Tachyglossus aculeatus
Gracilinanus agilis
Antechinus flavipes
Echinops telfairi
Procavia capensis
Talpa occidentalis
Sorex cinereus
Oryctolagus cuniculus
Ochotona princeps
Jaculus jaculus
Typhlomys cinereus
Dipodomys spectabilis
Aplodontia rufa
Order
C
Tachyglossus aculeatus
Gracilinanus agilis
Antechinus flavipes
Echinops telfairi
Procavia capensis
Talpa occidentalis
Sorex cinereus
Homo sapiens
Oryctolagus cuniculus
Ochotona princeps
Jaculus jaculus
Typhlomys cinereus
Mus musculus
Dipodomys spectabilis
Aplodontia rufa
0
2000
4000
6000
Nucleotide identity, %
60-70
70-80
80-90
> 90
Transposable elements
LINE
SINE
DNA
Other
SSRs
NEAT1Short 3'-end
Assembly gap
D
Identical region
0
1
2
3
4
5
6
7
kb

Supplementary Figure 3. Transposable elements in *NEAT1* orthologs.
A. Pairwise alignment of *NEAT1* orthologs carrying ERV-type TEs. Genomes are aligned along the phylogenetic tree. Only patches of similarity longer than 500bp are depicted.
B. Pairwise alignment of *NEAT1* archetypes, arranged along the phylogenetic tree. Patches of similarity longer than 100bp are depicted.
C. Graphical representation of the distribution of self-complementary regions in selected *NEAT1* orthologs. Each ortholog is aligned to itself, and reverse complementary regions are connected with lines, forming a visual 'cross' shape. Orthologs were selected to demonstrate the full diversity of possible distributions of self-complementary regions. Cases lacking such regions were not included.

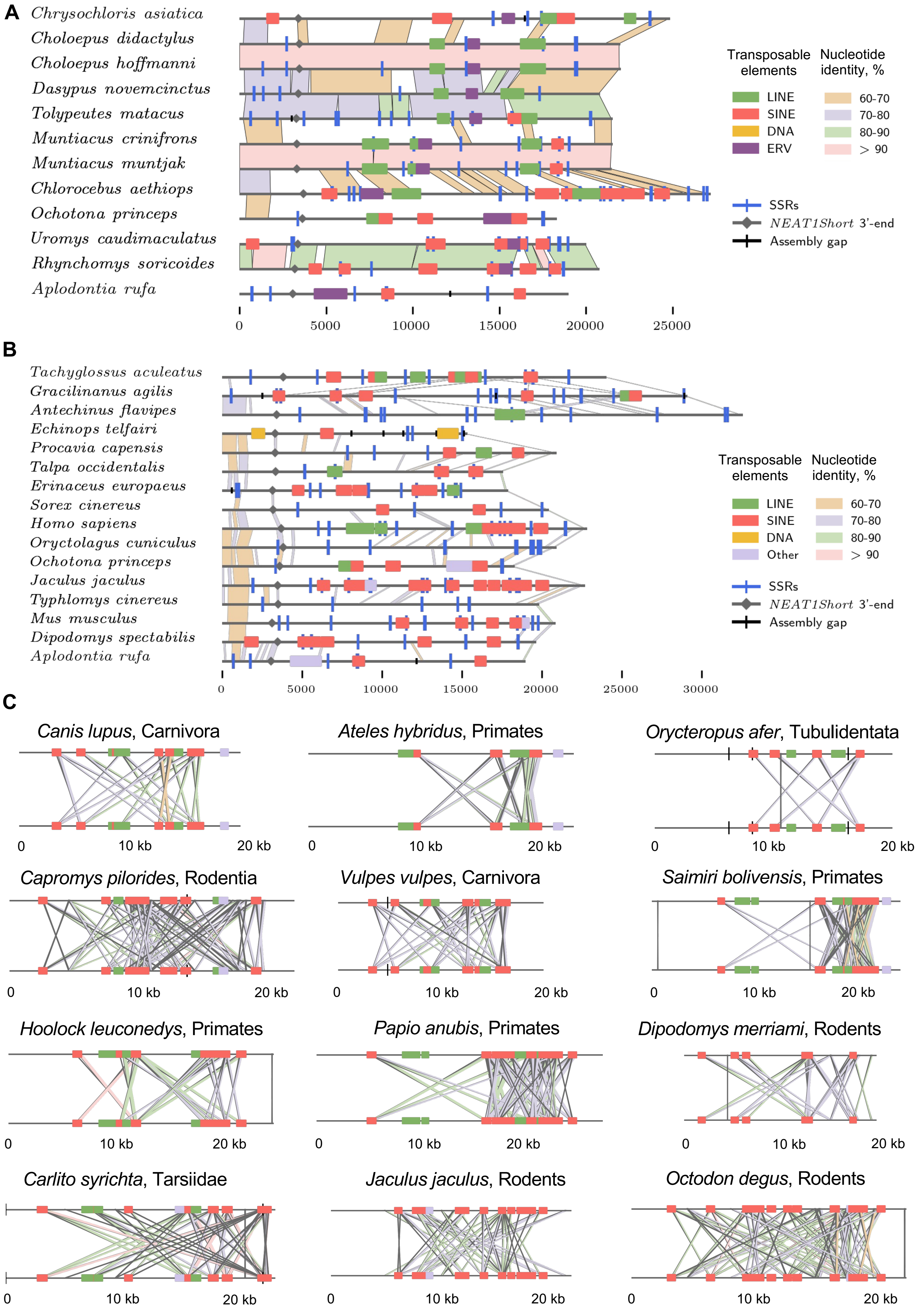

A
Chrysochloris asiatica
Choloepus didactylus
Choloepus hoffmanni
Dasypus novemcinctus
Tolypeutes matacus
Muntiacus crinifrons
Muntiacus muntjak
Chlorocebus aethiops
Ochotona princeps
Uromys caudimaculatus
Rhynchomys soricoides
Aplodontia rufa
0
5000
10000
15000
20000
25000
Transposable elements
LINE
SINE
DNA
ERV
Nucleotide identity, %
60-70
70-80
80-90
> 90
SSRs
NEAT1Short 3'-end
Assembly gap
B
Tachyglossus aculeatus
Gracilinanus agilis
Antechinus flavipes
Echinops telfairi
Procavia capensis
Talpa occidentalis
Erinaceus europaeus
Sorex cinereus
Homo sapiens
Oryctolagus cuniculus
Ochotona princeps
Jaculus jaculus
Typhlomys cinereus
Mus musculus
Dipodomys spectabilis
Aplodontia rufa
0
5000
10000
15000
20000
25000
30000
Transposable elements
LINE
SINE
DNA
Other
Nucleotide identity, %
60-70
70-80
80-90
> 90
SSRs
NEAT1Short 3'-end
Assembly gap
C
Canis lupus, Carnivora
Ateles hybridus, Primates
Orycteropus afer, Tubulidentata
Capromys pilorides, Rodentia
Vulpes vulpes, Carnivora
Saimiri bolivensis, Primates
Hoolock leuconedys, Primates
Papio anubis, Primates
Dipodomys merriami, Rodents
Carlito syrichta, Tarsiidae
Jaculus jaculus, Rodents
Octodon degus, Rodents
0
10 kb
20 kb

Supplementary Figure 4. Transposable elements in *NEAT1Short* and *MALAT1* orthologs.
A. Pairwise alignment of *NEAT1* orthologs carrying TEs in the *NEAT1Short* isoform. Species are ordered according to the phylogenetic tree. In the figure, only one *NEAT1* ortholog of *Canis lupus* from the eight different assemblies in our dataset is presented. The alignment of all eight orthologs of *Canis lupus* can be found in Suppl. Fig. 9.
B. *MALAT1* orthologs carrying TEs. Species are ordered according to the phylogenetic tree.

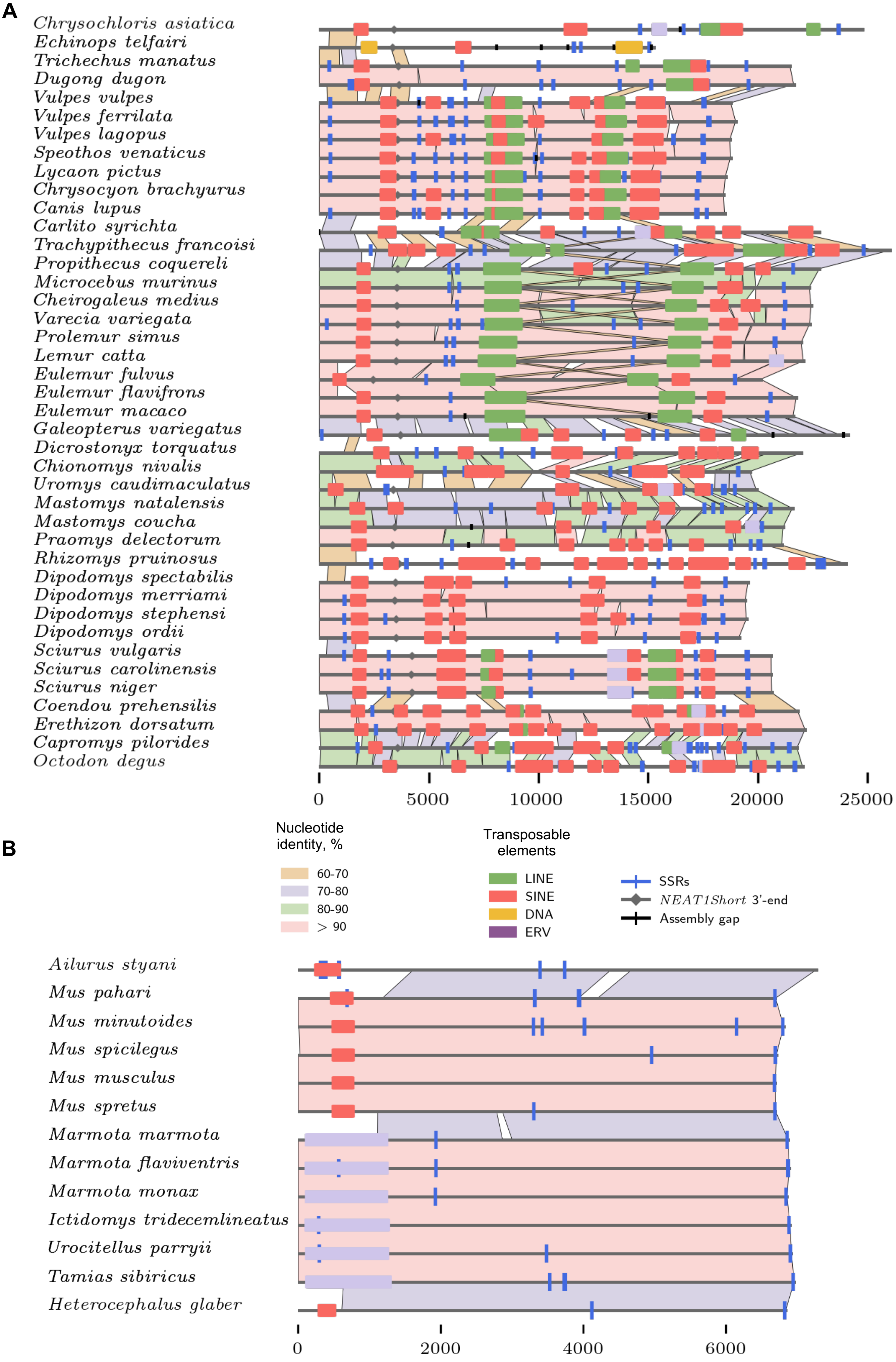

A
Chrysochloris asiatica
Echinops telfairi
Trichechus manatus
Dugong dugon
Vulpes vulpes
Vulpes ferrilata
Vulpes lagopus
Speothos venaticus
Lycaon pictus
Chrysocyon brachyurus
Canis lupus
Carlito syrichta
Trachypithecus francoisi
Propithecus coquereli
Microcebus murinus
Cheirogaleus medius
Varecia variegata
Prolemur simus
Lemur catta
Eulemur fulvus
Eulemur flavifrons
Eulemur macaco
Galeopterus variegatus
Dicrostonyx torquatus
Chionomys nivalis
Uromys caudimaculatus
Mastomys natalensis
Mastomys coucha
Praomys delectorum
Rhizomys pruinosus
Dipodomys spectabilis
Dipodomys merriami
Dipodomys stephensi
Dipodomys ordii
Sciurus vulgaris
Sciurus carolinensis
Sciurus niger
Coendou prehensilis
Erethizon dorsatum
Capromys pilorides
Octodon degus
0
5000
10000
15000
20000
25000
B
Nucleotide identity, %
60-70
70-80
80-90
> 90
Transposable elements
LINE
SINE
DNA
ERV
SSRs
NEAT1Short 3'-end
Assembly gap
Ailurus styani
Mus pahari
Mus minutoides
Mus spicilegus
Mus musculus
Mus spretus
Marmota marmota
Marmota flaviventris
Marmota monax
Ictidomys tridecemlineatus
Urocitellus parryii
Tamias sibiricus
Heterocephalus glaber
0
2000
4000
6000

Supplementary Figure 5. Nucleotide usage of *NEAT1* and *MALAT1* orthologs across mammalian orders. One species per family is depicted. The colour bar corresponds to mammalian orders, arranged along the phylogenetic tree.

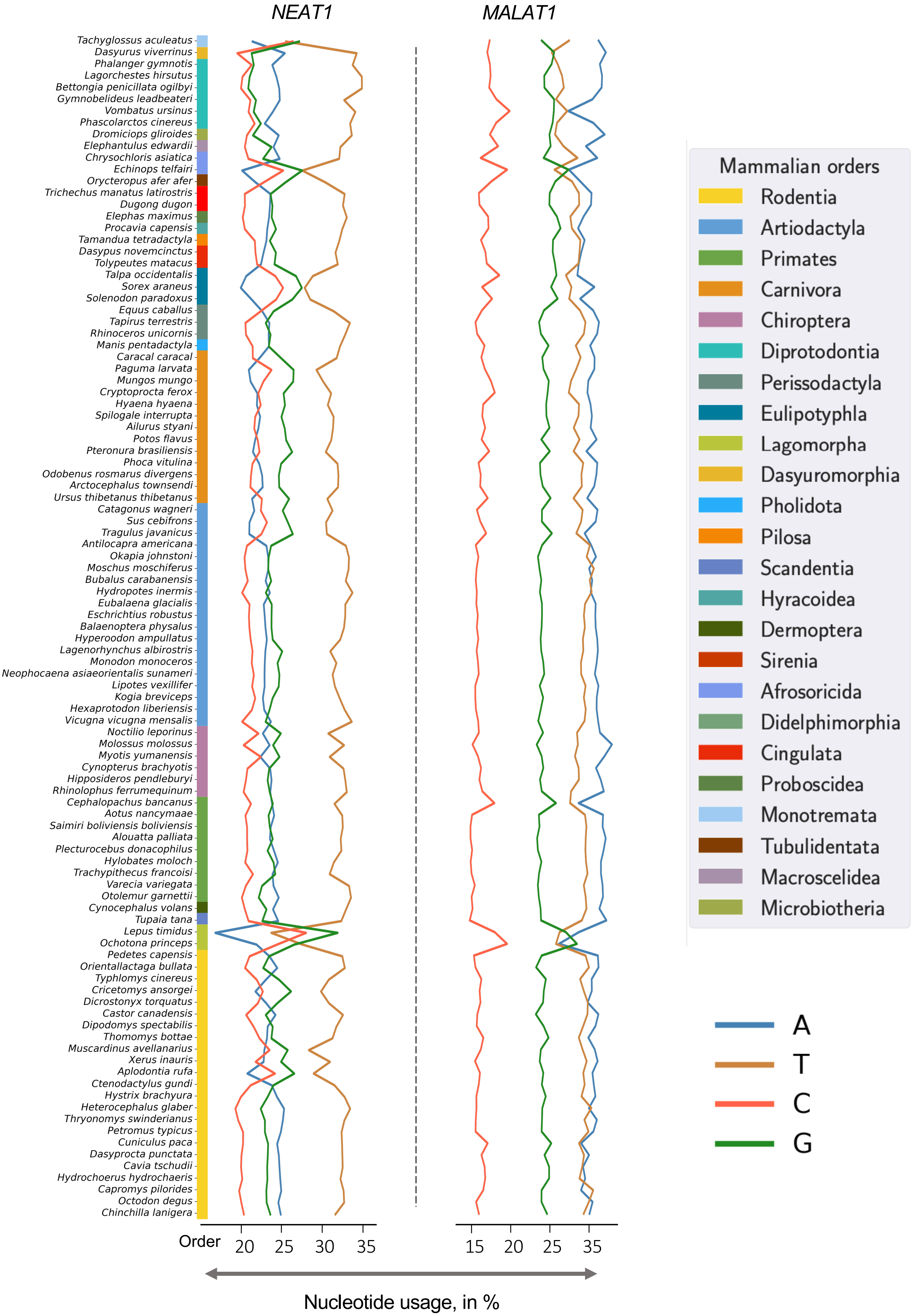

NEAT1
MALAT1
Tachyglossus aculeatus
Dasyurus viverrinus
Phalanger gymnotis
Lagorchestes hirsutus
Bettongia penicillata ogilbyi
Gymnobelideus leadbeateri
Vombatus ursinus
Phascolarctos cinereus
Dromiciops gliroides
Elephantulus edwardii
Chrysochloris asiatica
Echinops telfairi
Orycteropus afer afer
Trichechus manatus latirostris
Dugong dugon
Elephas maximus
Procavia capensis
Tamandua tetradactyla
Dasypus novemcinctus
Tolypeutes matacus
Talpa occidentalis
Sorex araneus
Solenodon paradoxus
Equus caballus
Tapirus terrestris
Rhinoceros unicornis
Manis pentadactyla
Caracal caracal
Paguma larvata
Mungos mungo
Cryptoprocta ferox
Hyaena hyaena
Spilogale interrupta
Ailurus styani
Potos flavus
Pteronura brasiliensis
Phoca vitulina
Odobenus rosmarus divergens
Arctocephalus townsendi
Ursus thibetanus thibetanus
Catagonus wagneri
Sus cebifrons
Tragulus javanicus
Antilocapra americana
Okapia johnstoni
Moschus moschiferus
Bubalus carabanensis
Hydropotes inermis
Eubalaena glacialis
Eschrichtius robustus
Balaenoptera physalus
Hyperoodon ampullatus
Lagenorhynchus albirostris
Monodon monoceros
Neophocaena asiaeorientalis sunameri
Lipotes vexillifer
Kogia breviceps
Hexaprotodon liberiensis
Vicugna vicugna mensalis
Noctilio leporinus
Molossus molossus
Myotis yumanensis
Cynopterus brachyotis
Hipposideros pendleburyi
Rhinolophus ferrumequinum
Cephalopachus bancanus
Aotus nancymaae
Saimiri boliviensis boliviensis
Alouatta palliata
Plecturocebus donacophilus
Hylobates moloch
Trachypithecus francoisi
Varecia variegata
Otolemur garnettii
Cynocephalus volans
Tupaia tana
Lepus timidus
Ochotona princeps
Pedetes capensis
Orientallactaga bullata
Typhlomys cinereus
Cricetomys ansorgei
Dicrostonyx torquatus
Castor canadensis
Dipodomys spectabilis
Thomomys bottae
Muscardinus avellanarius
Xerus inauris
Aplodontia rufa
Ctenodactylus gundi
Hystrix brachyura
Heterocephalus glaber
Thryonomys swinderianus
Petromus typicus
Cuniculus paca
Dasyprocta punctata
Cavia tschudii
Hydrochoerus hydrochaeris
Capromys pilorides
Octodon degus
Chinchilla lanigera
Order
20 25 30 35
15 20 25 35
Nucleotide usage, in %
Mammalian orders
Rodentia
Artiodactyla
Primates
Carnivora
Chiroptera
Diprotodontia
Perissodactyla
Eulipotyphla
Lagomorpha
Dasyuromorphia
Pholidota
Pilosa
Scandentia
Hyracoidea
Dermoptera
Sirenia
Afrosoricida
Didelphimorphia
Cingulata
Proboscidea
Monotremata
Tubulidentata
Macroscelidea
Microbiotheria
A
T
C
G

Supplementary Figure 6. Plot of nucleotide usage along the sequence for *NEAT1* archetypes. The length of the ortholog was binned into 5% segments, and the nucleotide usage of each bin was estimated. The average nucleotide usage is depicted with dashed lines.

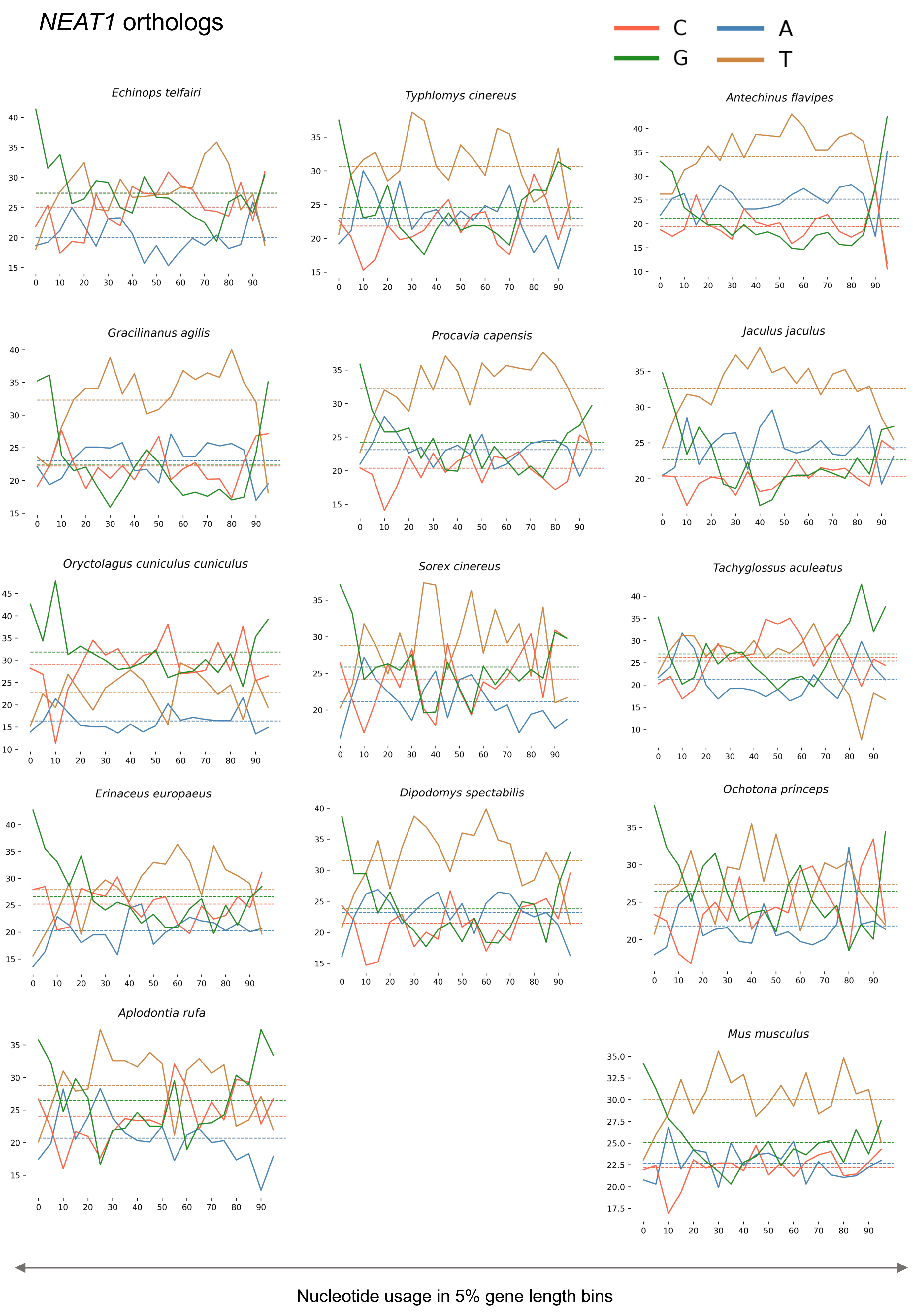

NEAT1 orthologs
C
A
G
T
Echinops telfairi
Typhlomys cinereus
Antechinus flavipes
Gracilinanus agilis
Procavia capensis
Jaculus jaculus
Oryctolagus cuniculus cuniculus
Sorex cinereus
Tachyglossus aculeatus
Erinaceus europaeus
Dipodomys spectabilis
Ochotona princeps
Aplodontia rufa
Mus musculus
Nucleotide usage in 5% gene length bins

Supplementary Figure 7. Plot of nucleotide usage along the sequence for *MALAT1* archetypes. The length of the ortholog was binned into 5% segments, and the nucleotide usage of each bin was estimated. The average nucleotide usage is depicted with dashed lines.

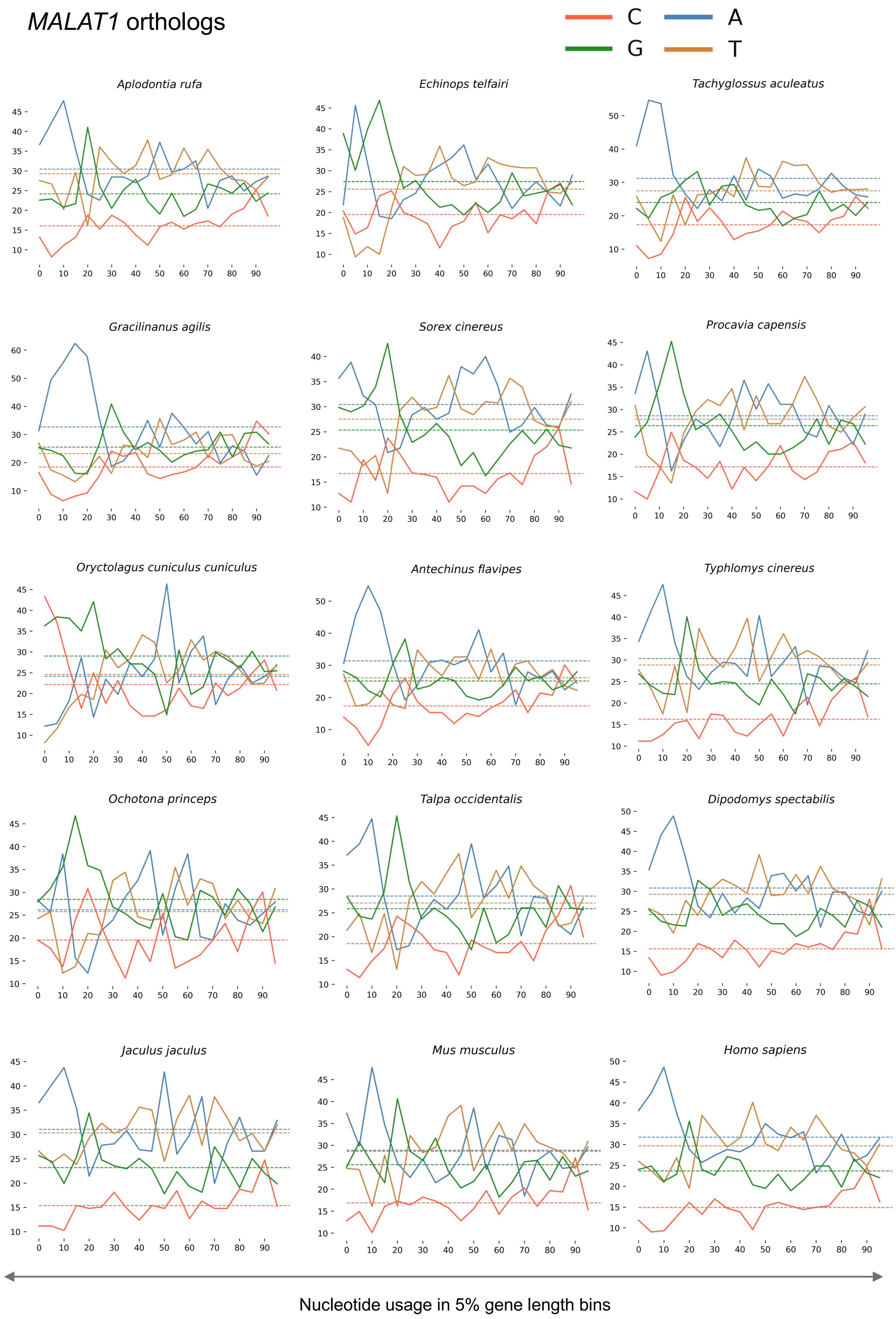
MALAT1 orthologs
C
A
G
T
Aplodontia rufa
Echinops telfairi
Tachyglossus aculeatus
Gracilinanus agilis
Sorex cinereus
Procavia capensis
Oryctolagus cuniculus cuniculus
Antechinus flavipes
Typhlomys cinereus
Ochotona princeps
Talpa occidentalis
Dipodomys spectabilis
Jaculus jaculus
Mus musculus
Homo sapiens
Nucleotide usage in 5% gene length bins

Supplementary Figure 8. Distribution of the most frequent hexamers and G-quadruplexes
A. Distribution of two groups of hexamers and G-quadruplexes in *NEAT1* archetypes. Each vertical line, in the colours described in the legend, marks the position of the identified element.
B. Distribution of the identified universal features in non-mammalian vertebrates. Each ortholog's length was divided into 10% bins, and the number of detected elements was summed per bin.
C. The most frequent species-specific hexamers. The depicted hexamers are listed on the left side of the ortholog map. Each vertical line, in the colours described in the legend, marks the position of the identified element. Ortholog maps are paired with the sequence complexity plot, highlighting drops in complexity caused by clustering of hexamer repeats. The mean value of sequence complexity is plotted with a dashed line.

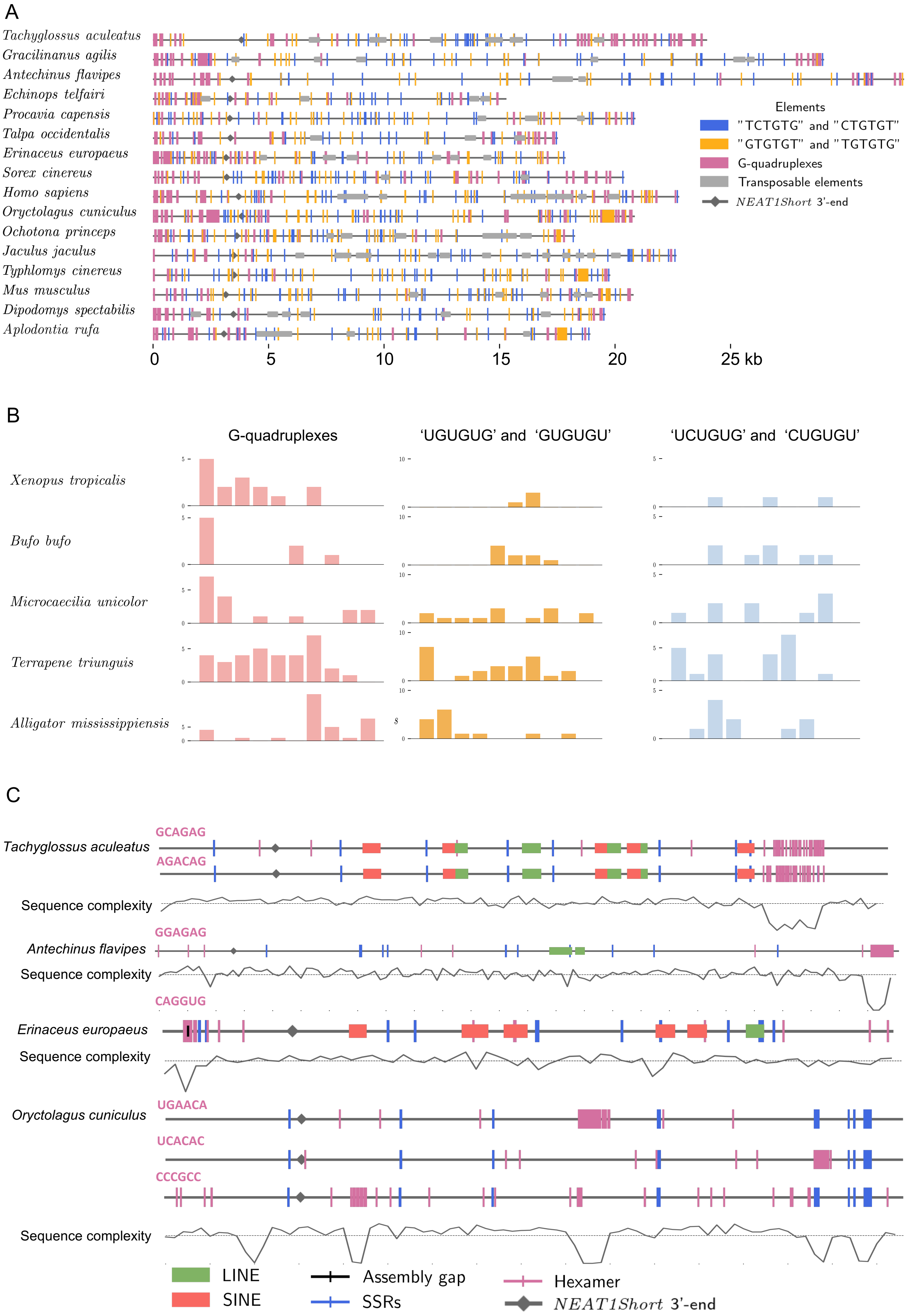

A
Tachyglossus aculeatus
Gracilinanus agilis
Antechinus flavipes
Echinops telfairi
Procavia capensis
Talpa occidentalis
Erinaceus europaeus
Sorex cinereus
Homo sapiens
Oryctolagus cuniculus
Ochotona princeps
Jaculus jaculus
Typhlomys cinereus
Mus musculus
Dipodomys spectabilis
Aplodontia rufa
0
5
10
15
20
25 kb
Elements
"TCTGTG" and "CTGTGT"
"GTGTGT" and "TGTGTG"
G-quadruplexes
Transposable elements
NEAT1Short 3'-end
B
G-quadruplexes
'UGUGUG' and 'GUGUGU'
'UCUGUG' and 'CUGUGU'
Xenopus tropicalis
Bufo bufo
Microcaecilia unicolor
Terrapene triunguis
Alligator mississippiensis
C
GCAGAG
Tachyglossus aculeatus
AGACAG
Sequence complexity
GGAGAG
Antechinus flavipes
Sequence complexity
CAGGUG
Erinaceus europaeus
Sequence complexity
UGAACA
Oryctolagus cuniculus
UCACAC
CCCGCC
Sequence complexity
LINE
SINE
Assembly gap
SSRs
Hexamer
NEAT1Short 3'-end

Supplementary Figure 9. Pairwise alignments of *NEAT1* orthologs from different assemblies of the same species. On the left side, the ANI value of the pairwise comparison is indicated.

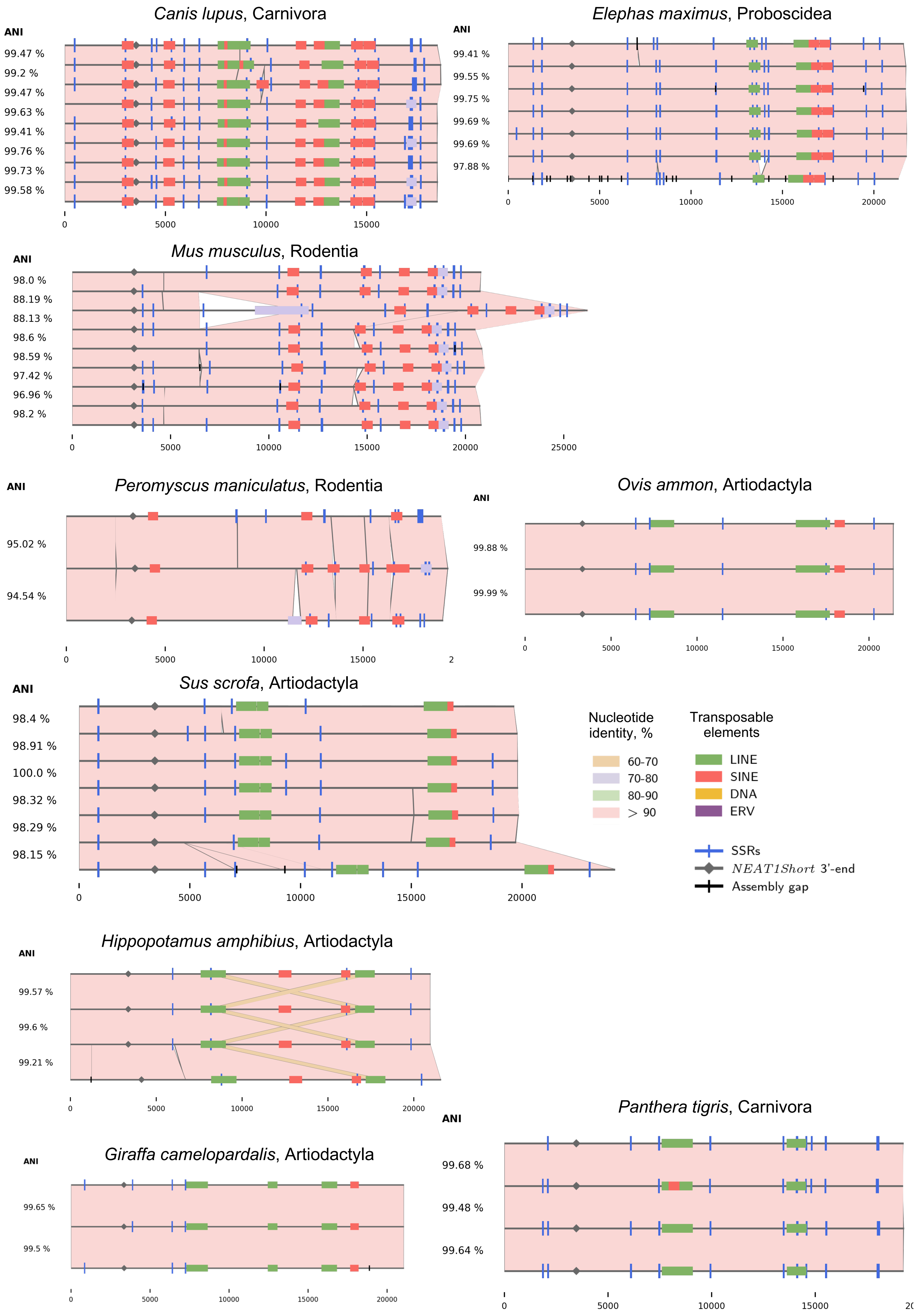

Canis lupus, Carnivora
ANI
99.47 %
99.2 %
99.47 %
99.63 %
99.41 %
99.76 %
99.73 %
99.58 %
Elephas maximus, Proboscidea
ANI
99.41 %
99.55 %
99.75 %
99.69 %
99.69 %
97.88 %
Mus musculus, Rodentia
ANI
98.0 %
88.19 %
88.13 %
98.6 %
98.59 %
97.42 %
96.96 %
98.2 %
Peromyscus maniculatus, Rodentia
ANI
95.02 %
94.54 %
Ovis ammon, Artiodactyla
ANI
99.88 %
99.99 %
Sus scrofa, Artiodactyla
ANI
98.4 %
98.91 %
100.0 %
98.32 %
98.29 %
98.15 %
Nucleotide identity, %
60-70
70-80
80-90
> 90
Transposable elements
LINE
SINE
DNA
ERV
SSRs
NEAT1Short 3'-end
Assembly gap
Hippopotamus amphibius, Artiodactyla
ANI
99.57 %
99.6 %
99.21 %
Giraffa camelopardalis, Artiodactyla
ANI
99.65 %
99.5 %
Panthera tigris, Carnivora
ANI
99.68 %
99.48 %
99.64 %

Supplementary Figure 10. Instances of detected excised SINE elements. Pairwise alignment of *Macaca*, *Mustela* and *Microtus* genera. Regions with deleted SINE elements are highlighted by a dashed frame. Bootstrap support values are indicated.

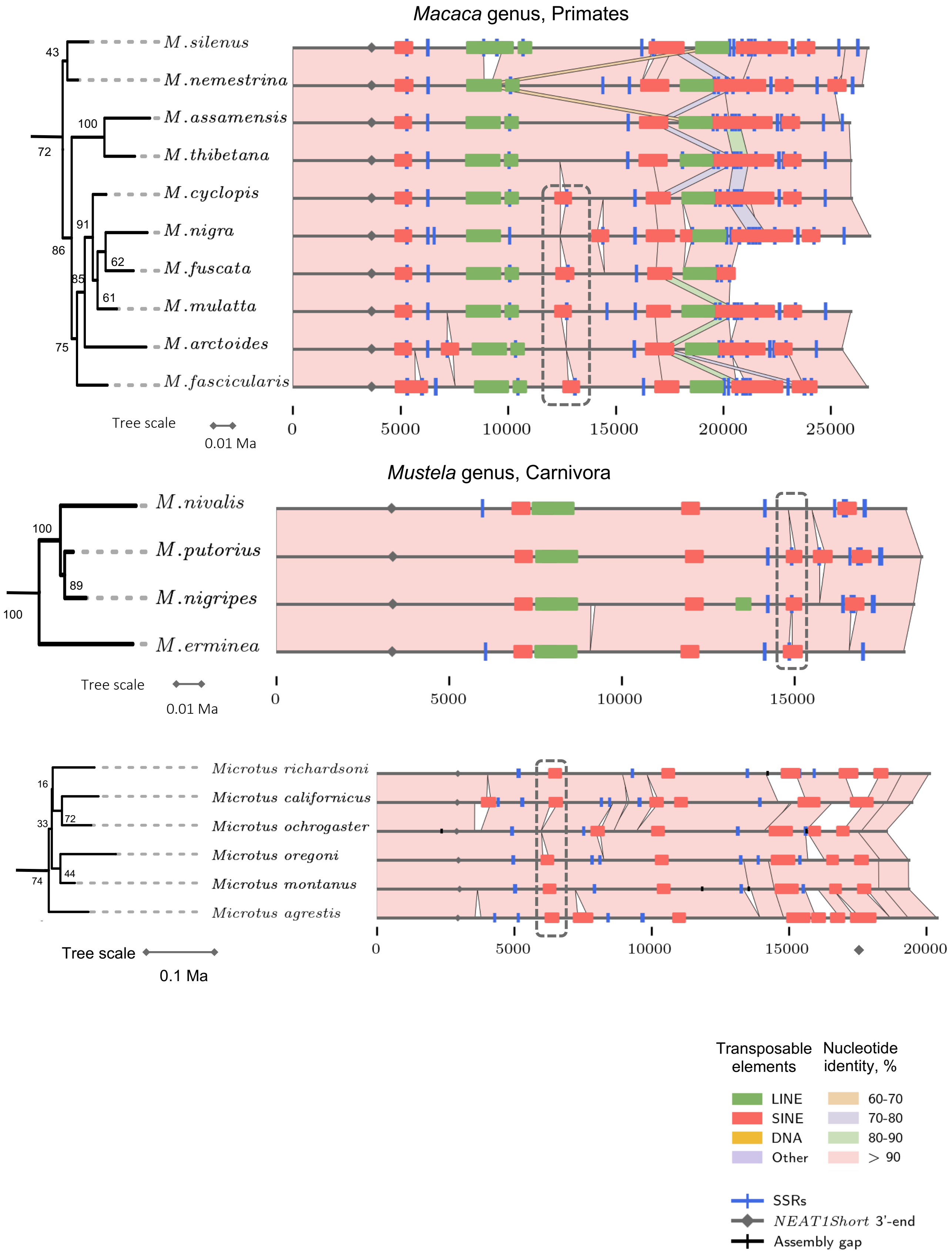

Macaca genus, Primates
M.silenus
M.nemestrina
M.assamensis
M.thibetana
M.cyclopis
M.nigra
M.fuscata
M.mulatta
M.arctoides
M.fascicularis
43
100
72
91
86
62
85
61
75
Tree scale
0.01 Ma
0 5000 10000 15000 20000 25000
Mustela genus, Carnivora
M.nivalis
M.putorius
M.nigripes
M.erminea
100
89
100
Tree scale
0.01 Ma
0 5000 10000 15000
Microtus richardsoni
Microtus californicus
Microtus ochrogaster
Microtus oregoni
Microtus montanus
Microtus agrestis
16
72
33
44
74
Tree scale
0.1 Ma
0 5000 10000 15000 20000
Transposable elements
LINE
SINE
DNA
Other
Nucleotide identity, %
60-70
70-80
80-90
> 90
SSRs
NEAT1Short 3'-end
Assembly gap
